# S-adenosylhomocysteine hydrolase-like protein 1 (AHCYL1) inhibits lung cancer tumorigenesis by regulating cell plasticity

**DOI:** 10.1186/s13062-023-00364-y

**Published:** 2023-03-05

**Authors:** Melina Muñoz-Bernart, Nicolás Budnick, Araceli Castro, Malena Manzi, María Eugenia Monge, Julieta Pioli, Sebastián Defranchi, Gustavo Parrilla, Juan Pablo Santilli, Kevin Davies, Joaquín M. Espinosa, Ken Kobayashi, Carlos Vigliano, Carolina Perez-Castro

**Affiliations:** 1 Instituto de Investigación en Biomedicina de Buenos Aires (IBioBA) – CONICET, Partner Institute of the Max Planck Society, Buenos Aires, Argentina; 2 Instituto de Medicina Traslacional, Trasplante y Bioingeniería (IMeTTyB), Universidad Favaloro-CONICET, Solís 453, C1078AAI Buenos Aires, Argentina; 3 Centro de Investigaciones en Bionanociencias (CIBION), Consejo Nacional de Investigaciones Científicas y Técnicas (CONICET), Godoy Cruz 2390, C1425FQD Ciudad de Buenos Aires, Argentina; 4 Departamento de Fisiología, Biología Molecular y Celular, Facultad de Ciencias Exactas y Naturales, Universidad de Buenos Aires, Intendente Güiraldes, 2160 C1428EGA Buenos Aires, Argentina; 5 Consejo Nacional de Investigaciones Científicas y Técnicas (CONICET), Departamento de Desarrollo Analítico y Control de Procesos, Instituto Nacional de Tecnología Industrial, Av. General Paz 5445, B1650WAB Buenos Aires, Argentina; 6 Servicio de Cirugía Torácica, Hospital Universitario de la Fundación Favaloro, Av. Belgrano 1746, C1093AAS Buenos Aires, Argentina; 7 Servicio de Anatomía Patológica, Hospital Universitario de la Fundación Favaloro, Av. Belgrano 1746, C1093AAS Buenos Aires, Argentina; 8 Linda Crnic Institute for Down Syndrome, University of Colorado Anschutz Medical Campus, Aurora, CO USA; 9 Department of Pharmacology, University of Colorado Anschutz Medical Campus, Aurora, CO USA; 10 Department of Molecular, Cellular and Developmental Biology, University of Colorado Boulder, Boulder, CO USA; 11 Laboratorio de Agrobiotecnología, Instituto de Biodiversidad y Biología Experimental Aplicada (IBBEA-CONICET-UBA), Facultad de Ciencias Exactas y Naturales, Universidad de Buenos Aires, Buenos Aires, Argentina

**Keywords:** IRBIT, Lung cancer stem cells (LCSC), Biomarker, NSCLC

## Abstract

**Background:**

Lung cancer is one of the most frequently diagnosed cancers characterized by high mortality, metastatic potential, and recurrence. Deregulated gene expression of lung cancer, likewise in many other solid tumors, accounts for their cell heterogeneity and plasticity. S-adenosylhomocysteine hydrolase-like protein 1 (AHCYL1), also known as Inositol triphosphate (IP(3)) receptor-binding protein released with IP(3) (IRBIT), plays roles in many cellular functions, including autophagy and apoptosis but AHCYL1 role in lung cancer is largely unknown.

**Results:**

Here, we analyzed the expression of AHCYL1 in Non-Small Cell Lung Cancer (NSCLC) cells from RNA-seq public data and surgical specimens, which revealed that AHCYL1 expression is downregulated in tumors and inverse correlated to proliferation marker Ki67 and the stemness signature expression. AHCYL1-silenced NSCLC cells showed enhanced stem-like properties in vitro, which correlated with higher expression levels of stem markers POU5F1 and CD133. Also, the lack of AHCYL1 enhanced tumorigenicity and angiogenesis in mouse xenograft models highlighting stemness features.

**Conclusions:**

These findings indicate that AHCYL1 is a negative regulator in NSCLC tumorigenesis by modulating cell differentiation state and highlighting AHCYL1 as a potential prognostic biomarker for lung cancer.

**Supplementary Information:**

The online version contains supplementary material available at 10.1186/s13062-023-00364-y.

## Background

One of the most frequent subtypes of lung cancer (LC) is non-small cell lung cancer (NSCLC), which includes adenocarcinoma, squamous cell carcinoma, and large cell carcinoma [1]. Despite available treatments, a high percentage of patients present recurrence and high mortality [1].

Like many other solid tumors, LC presents severe deregulation of gene expression associated with elevated tumor resistance and relapse [2–4]. Lung cancer stem-like cells (LCSCs) have stem-like properties and contribute to tumor cell plasticity and heterogeneity, but the underlying molecular mechanisms are not fully understood, although core pluripotency factors and epigenetic regulators are elevated [5, 6].

Previously, we have identified the gene S-adenosylhomocysteine hydrolase-like protein 1 (AHCYL1), also known as IRBIT (Inositol triphosphate (IP3) receptor-binding protein released with IP3) as a potential gene regulated in cancer stem cells using a bioinformatics tool [7–9]. AHCYL1 is a multifaceted and ubiquitously expressed protein and is involved in intracellular calcium and pH regulation, dNTPs availability, and promotion of apoptosis [10–14]. However, the role of AHCYL1 during mammalian cell plasticity and cancer progression is still poorly understood. AHCYL1 expression is downregulated in drug-resistant cancer cell lines and also in human ovarian cancer [15]. Also, low expression of AHCYL1 was associated with poor prognosis and recurrence in gastric cancer [16]. A recent report indicated that AHCYL1 negatively regulates autophagy in U20S and Hela cancer cells [17, 18]. In addition, AHCYL1 can inhibit S-Adenosylhomocysteine hydrolase (AHCY), a key enzyme that reversibly catalyzes the hydrolysis of S-Adenosylhomocysteine (SAH) to Homocysteine (Hcy) and Adenosine [10, 19], and Ribonucleotide reductase (RNR), which is required for cell cycle progression [12, 20].

Also, metabolic reprogramming has substantial role in tumor initiation and maintenance [21]. High methionine cycle activity and transmethylation rates were recently associated with tumor initiation capacity in LC [22–24]. Nevertheless, the role of methionine metabolism in tumorigenesis and cell heterogeneity in LC is poorly characterized.

All this evidence points to AHCYL1 as having a relevant role in LC and we decided to study the expression of AHCYL1 by performing an integrated analysis including bioinformatics, immunohistochemistry, and in vivo experiments. Our research determined that AHCYL1 links metabolism, cell differentiation state, and tumorigenesis in LC. Moreover, we present results highlighting AHCYL1 as a for potential prognostic biomarker.

## Methods

### Tissue samples

Details are in Extended Protocol (see Additional file 5). This is a retrospective study of surgical samples from patients undergoing surgery at the Hospital Universitario de la Fundación Favaloro between November 2009 and December 2020. Informed consent was collected according to the hospital’s institutional review board (in compliance with the October 2013 Helsinki Declaration). The research protocol was approved by the Bioethics Committee of the Fundación Favaloro DDI (1473) 0719.

The histologic classification published by the World Health Organization (WHO) for tumors of the lung was applied [25].

None of the patients had prior chemo or radiation therapy. Sections were stained with hematoxylin and eosin to assess the histologic grade of neoplasms, which was classified according to the criteria of the College of American Pathologists (CAP) [26].

The AJCC and UICC TNM staging system was used for Pathologic Stage Classification for both adenocarcinoma and other non-small cell lung cancer [27].

Survival and recurrence were calculated from the time of lung resection to the date of the last consultation or the date of the patient's death.

### Immunohistochemistry staining and analyses

Details are in Extended Protocol (﻿see Additional file 5). Briefly, staining was performed using an automated immune stainer (BenchMark GX, Ventana Medical Systems/Roche, Tucson, AZ, USA).

The intensity of the AHCYL1 staining was scored semi-quantitatively from 0 to 3 + , as follows: 0, no staining; 1, weak staining; 2, moderate staining and 3 or more, intense staining. The Ki67 antigen was used as an indicator of entry into the cell cycle of the neoplastic cells, quantifying according to the proportion of neoplastic cells with nuclei with positive staining at high magnification (400x). In all cases, the evaluations of the samples were performed independently by two pathologists (KD and JPS) blinded to the clinical characteristics of the patients. Disagreements regarding histological diagnoses and immunohistochemistry evaluation were discussed and resolved by consensus with a third pathologist (CV). Antibodies are listed in Additional file 1: Table S1.

### Bioinformatics analysis on patient datasets

RNA expression and clinical data from “The Cancer Genome Atlas” (TCGA) lung adenocarcinoma (LUAD) and lung squamous cell carcinoma (LUSC) datasets were obtained from UCSC Xena browser tool (RRID:SCR_018938) [28]. For *Ki67*, *AHCYL1* expression data and pluripotency index (mRNA expression based and epigenetically regulated based) [29], Spearman´s correlation analysis was used. The GraphPad prism 8 software (RRID:SCR_002798) was used on LUAD and LUSC datasets. The Kaplan–Meier survival plot was generated using the KM Plotter tool (https://kmplot.com/analysis, RRID: SCR_018753); Mantel-Cox test (log-rank test), was used to compare the survival of two subgroups.

### Cell culture

HEK 293T (RRID:CVCL_0063), A549 (RRID:CVCL_0023) and H1299 (RRID:CVCL_0060) cell lines were acquired from the American Type Culture Collection (ATCC), either directly or from colleagues, kept frozen at liquid Nitrogen after received and used in culture for a maximum of 4 months. ATCC cell lines were characterized by Short Tandem Repeat (STR) profiling and Mycoplasm contamination was evaluated monthly by PCR. All cell lines were cultured in complete Dulbecco's Modified Eagle Medium (DMEM) supplemented with 10% fetal bovine serum (FBS), penicillin 100U.ml-1 /streptomycin 100 µg.ml-1 and L-glutamine 2 mM in 5% CO2 humidified atmosphere at 37 °C.

For sphere induction, LC cells were grown to 90% confluence, trypsinized, and plated in stem cell (SC) medium in ultra-low adhesion multi-well plates (Corning) [9]. After 7 days, the number of spheres was quantified using 10× magnifications under a phase contrast microscope (Carl-Zeiss, AxioObserverZ1).

### Quantitative real-time PCR

Total RNA, cDNA and Real-time PCR was performed as described previously and calculated with the 2^−ΔΔCT^ method [9, 30]. Primers are listed in Additional file 2: Table S2.

### Western blotting

Western blot (WB) analysis was performed as described previously [9]. Cells lysates were prepared in 2 × Laemmly buffer and separated in sodium dodecyl sulfate polyacrylamide gel electrophoresis (SDS-PAGE). Membranes were incubated with specific primary antibodies followed by incubation with HRP-conjugated secondary antibodies (Bio-Rad Laboratories, Hercules, CA, USA). Developing was performed with the SuperSignal West Dura kit according to manufacturer’s instruction (Pierce Biotechnology, Waltham, MA, USA) using G:BOX-CHEMI-XT4 (Synoptics Ltd., Cambridge, United Kingdom). Antibodies are listed in Additional file 1: Table S1.

### Flow cytometry

Cells were incubated with anti-CD133/1 (AC133)-PE conjugate antibody (130-080-801, RRID:AB_244342) (MiltenyiBiotec) [9]. Data was acquired on a FACSCantoII instrument (BD Biosciences) and analyzed using Floreada.io software (https://floreada.io/analysis). The isotype control sample was used to establish a gate in the PE channel. Cells showing signal for CD133 above the gate established were deemed to be CD-positive cells.

### shRNA knockdown

Knockdown cell lines were generated as described previously [9]. Knockdown efficiency was confirmed by qRT-PCR and Western Blotting and periodically checked. Target sequences are listed in Additional file 3: Table S3.

### Limiting dilution assay

Performed as described previously [9]. Tumor-initiating cell frequency and *p*-values were calculated using Extreme Limiting Dilution Analysis (ELDA) software (RRID:SCR_018933) [31].

### In vivo assay

Animals were housed with access to food and water ad libitum in ventilated mouse cages (1–5 mice per cage) at the IBioBA Animal Services Facility. Experiments were performed according to ARRIVE guidelines [32] and approved by the Ethical Committee on Animal Care and Use (CICUAL), University of Buenos Aires, Argentina (No. 110-2019) and IBioBA-CONICET (2021-03-PC). *For the mouse xenograft model* 2 × 10^6^ A549 or H1299 cells either A549-NT (non-targeted) or KD-AL1-4-cells (stably knockdown of AHCYL1 4), were subcutaneously injected into the right flank of *NODscid* mice (Jackson Laboratory, ME, USA, RRID:IMSR_JAX:001,303) of 6–8 weeks of age. Tumor growth and total animal weight were measured weekly. After 7–8 weeks, tumors were resected for analysis. Tumor volume was calculated using the following formula: 0.5 × length × width^2^ (mm^3^) [33]. *For the* in vivo* angiogenesis assay*, 10^6^ cells of each A549-NT or KD-AL1-4-cells were harvested in DMEM-Trypan Blue (9:1) and were intradermally injected (27G needle) in the right flank of male NOD*scid* mice and the vehicle was injected in the left flank. Mice were randomly selected for each group (treatment). At 7 days, photographs were taken under stereo microscope (Stemi 305, Carl Zeiss, Oberkochen, Germany) using ZEN software (Carl ZEISS, Oberkochen, Germany, RRID:SCR_018163), measured using ImageJ software and calculated as (number of vessels cells side − number of vessels vehicle side)/Area. Data analysis was performed by two independent blinded observers.

### Cell culture for SAM and SAH extraction and detection by LC–MS

A protocol for cell culture and metabolite extraction was based on a previous report [34]. Sample generation was repeated 9 times; 4 samples for each cell with one blank in each experiment within 3 weeks. NT and KD-AHCYL 1–4 cells with cell passage number from 8 to 17 were used (more details in the Extended Protocol, ﻿see Additional file 5).

### UPLC-QTOF-MS analyses

SAM (S-Adenosyl methionine) and SAH intracellular levels were evaluated through a semi-targeted UPLC-QTOF-MS-based strategy. The analytical method is described in Extended Protocol. Peak areas were normalized to the cell number and were used to perform semi quantitative analyses.

### Statistical analyses

Kolmogorov–Smirnov test was performed for quantitative variables distribution. Normal distributions were displayed as mean and standard deviation. Non-Gaussian distributions were presented as medians and interquartile range 25–75%. Categorical variables were reported as percentages of the total and were analyzed using Fisher's Chi-square test. For the analysis of normal distribution, the t-test or ANOVA for independent samples was used and for non-parametric distribution, Mann Whitney or Kruskal–Wallis’ tests were used.

Univariate Cox regression for the survival rate is according to demographic, clinical, surgical, and pathological variables, Ki67 staining, and intensity of immunohistochemically staining for AHCYL1. Hazard ratio (HR) and confidence intervals (95% CI) were reported for each variable. All *p*-values reported were two-tailed and *p* < 0.05 was considered statistically significant. Statistical analysis was performed with SPSS 17.0 software (SPSS Inc, Chicago, Illinois, RRID:SCR_002865).

For metabolites, Mann–Whitney U tests were used for statistical analysis [34].

Progenitor frequencies from limiting dilution assays were determined using the software tool (ELDA) [31].

Statistical details of experiments can be found in the figure legends and results section.

## Results

### *AHCYL1* is downregulated in high-grade NSCLC tumors

**Fig. 1:**
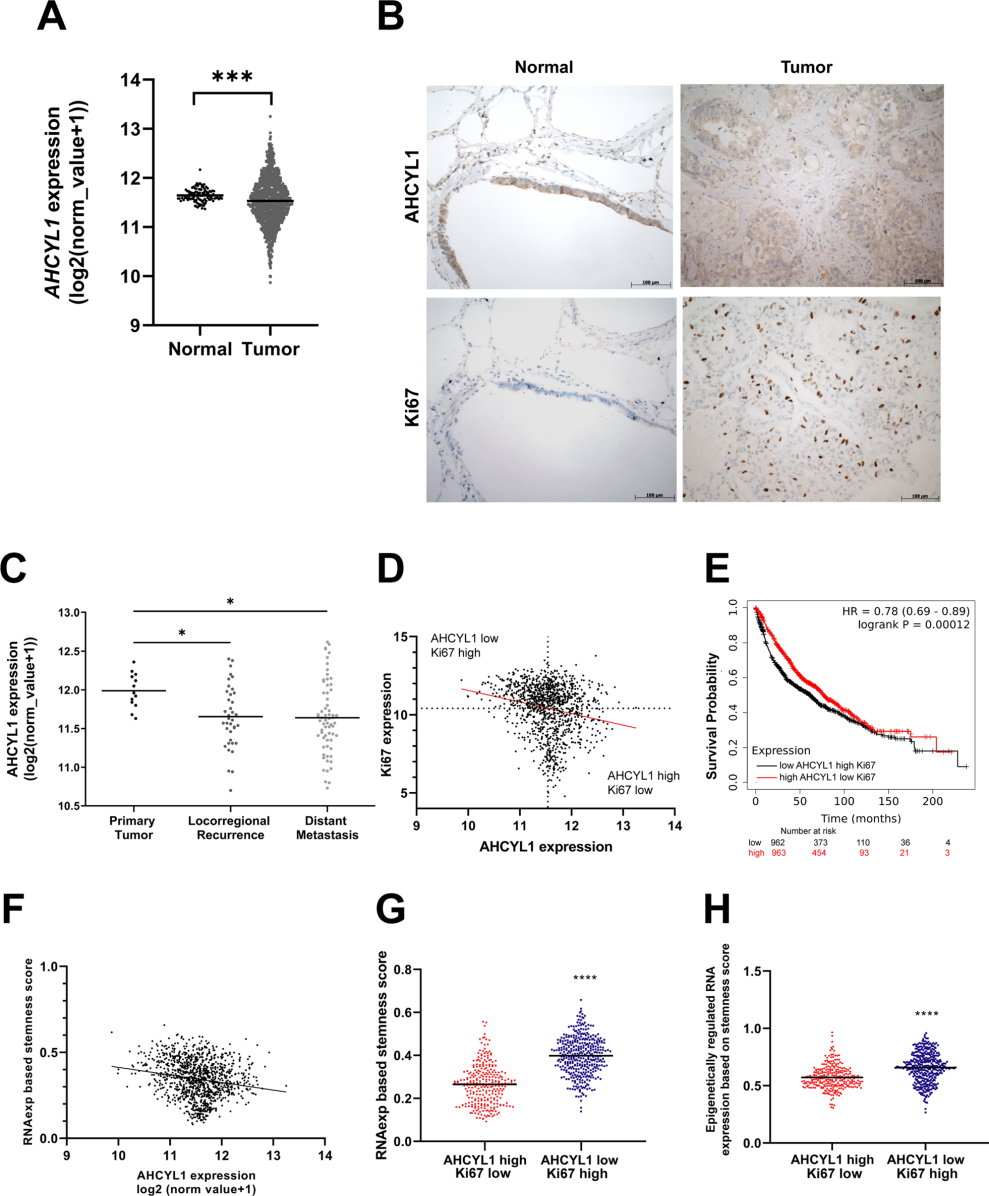
Transcriptomic analysis and immunohistochemistry assay of *AHCYL1* in human lung cancer. **A** Column scatter plot showing the normalized (log2 (normcount + 1)) expression of *AHCYL1* mRNA in normal tissue (n = 110) versus primary tumor (n = 1016) (Mann–Whitney Test, *p* = 0.0008). **B** IHC analysis of representative tissue samples from normal lung and lung adenocarcinoma tumor stained with an anti-AHCYL1 and Ki67antibodies. Scale bars = 100 µm. Original magnification × 200. **C** Column scatter plot showing the normalized (log2 (normcount + 1)) expression of *AHCYL1* mRNA in primary tumors (n = 13) vs. corresponding recurrence (n = 38) (*p* = 0.0403) vs. distant metastasis (n = 67) (*p* = 0.0209) (One-Way ANOVA, Tukey’s multiple comparisons test). **D** Spearman’s correlation analysis o *AHCYL1* expression vs. *Ki67* expression (n = 1105; *p* < 0.0001). **E** Kaplan–Meier for patients with Low *AHCYL1*-High *Ki67* versus High *AHCYL1*-Low *Ki67* analyzed with Mantel-Cox test (n = 1925; *p* = 0.00012). **F** Spearman’s correlation and linear regression analysis of *AHCYL1* expression versus stemness score (n = 1105; *p* < 0.0001). **G** High *AHCYL1* Low *Ki67* (n = 279) versus Low *AHCYL1* High *Ki67* (n = 381) stemness index (RNA expression based) (unpaired T test, *p* < 0.0001). **H** High *AHCYL1* Low *Ki67* (n = 279) versus Low *AHCYL1* High *Ki67* (n = 381) stemness index (epigenetically regulated RNA expression based) (unpaired T test with Welch correction, *p* < 0.0001). *p < 0.05, ***p < 0.001, ****p < 0.0001

The transcriptomic meta-analysis in normal tissue and NSCLC samples revealed that *AHCYL1* expression is significantly enriched in normal samples compared to LC (Fig. 1A). Particularly, *AHCYL1* shows a significantly lower expression in recurrences and metastases compared to primary biopsies (Fig. 1C) in LUAD samples, suggesting that adenocarcinoma with higher metastatic potential show lower *AHCYL1* expression.

The association analysis of *AHCYL1* and a tumor marker *Ki67* [35, 36] mRNA expression revealed a negative correlation (Fig. 1D). The patient samples classified into subgroups “high *AHCYL1* – low *Ki67*” and “low *AHCYL1*–high *Ki67*” were analyzed with the Kaplan Meier method, which showed that low *AHCYL1* and high *Ki67* patients exhibited worse prognosis (Fig. 1E).

Elevated pluripotency gene expression characterizes cell plasticity [3]. An analysis of Cis Regulatory Module of *AHCYL1* gene using the INSECT bioinformatics tool revealed potential binding sites for POU5F1 (POU Class 5 Homeobox 1) and SOX2 (SRY-Box Transcription Factor 2) [7–9], suggesting co-regulation by both core transcriptional factors of stem cells (Additional file 4: Fig. S1). Therefore, we evaluated the association between *AHCYL1* expression and cell pluripotency in LC. A stemness score index calculated for each sample showed to be statistically significant and negatively correlated between *AHCYL1* expression and the stemness score (Fig. 1F) [29]. We also calculated pluripotency traits associated with oncogenic dedifferentiation based on RNA expression and RNA epigenetic regulation obtaining the highest stemness score for samples with low *AHCYL1* and high *Ki67* (Fig. 1G, H).

**Fig. 2:**
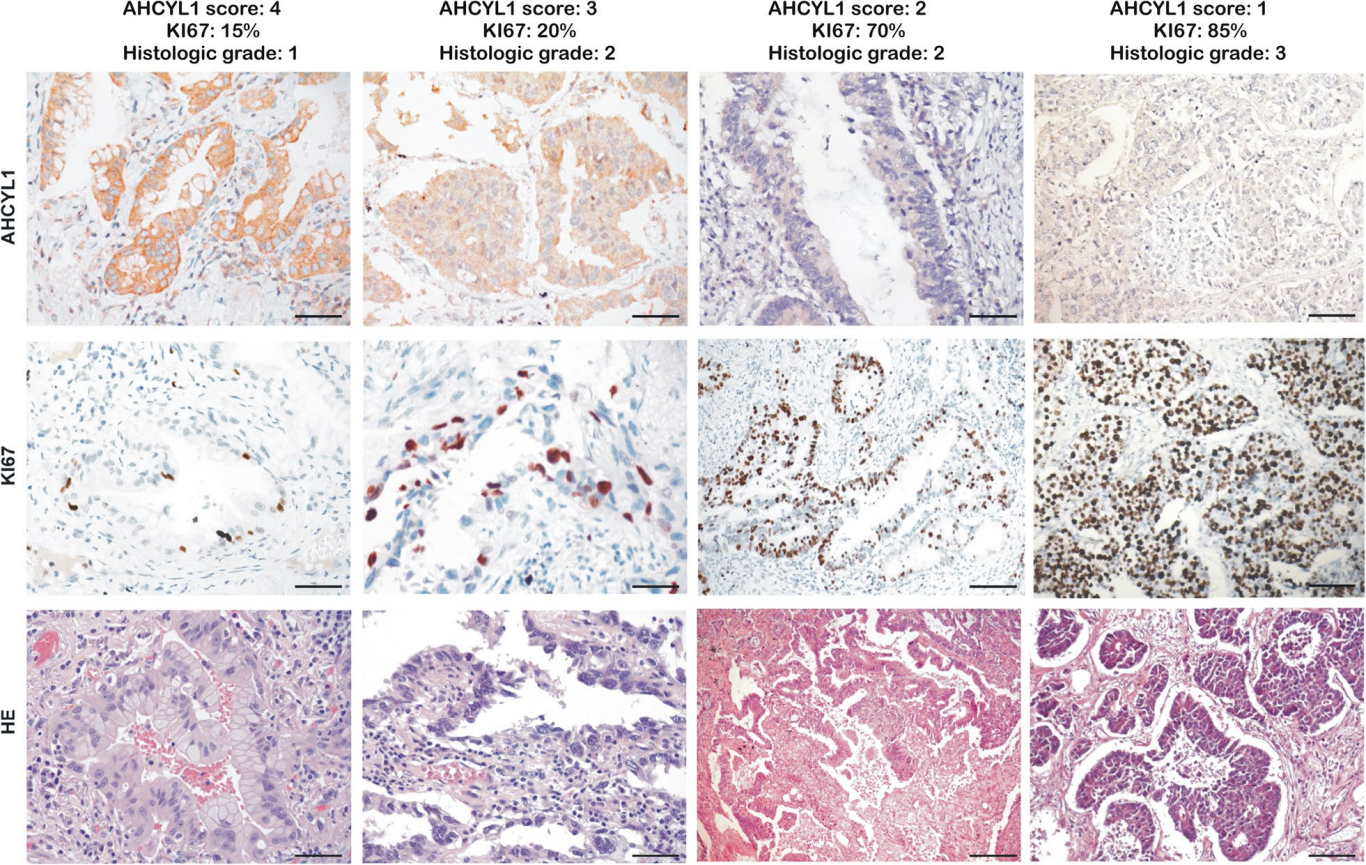
IHC staining of representative tissue samples with different types and grades of lung tumor. Sample sections stained with an anti–AHCYL1 antibody (top), anti-Ki67 antibody (middle) and Hematoxylin–Eosin (HE, bottom) for histologic grade score (n = 20). Scale bar, 100 µm

To assess the clinical relevance of these findings, AHCYL1 protein distribution and accumulation were analyzed by immunohistochemistry (IHC) staining in six normal tissues and 26 patient cases with non-small cell lung cancer: 20 (76.9%) corresponded to adenocarcinoma and six (23.1%) to tumors of other origins (three squamous, two neuroendocrine, and one large cell). In addition, the evaluation of other clinical and histological parameters was considered for analysis (see Additional file 5 and Additional file 6: Table S4). The specific staining of AHCYL1 was observed as heterogeneous, primarily in the cytoplasm, but also within the nucleus (Fig. 1B). In normal control tissues, strong AHCYL1 labeling was observed associated mainly at the epithelial lining of the distal airways (bronchioles) (Fig. 1B), a site proposed for the origin of preneoplastic lesions in adenocarcinoma cancer with the absence or very isolated signals from Ki67 staining [37]. One adenocarcinoma yielded an AHCYL1 staining score equal to 1 (5%); four cases exhibited score 2 (20%); nine cases score 3 (45%); and six cases score 4 (30%) (Additional file 6: Table S4). Remarkably, intense AHCYL1 labeling was observed in samples with well-differentiated cells. In contrast, images with poorly differentiated cells with worse prognoses showed a weak or low intensity of AHCYL1 staining associated with intense Ki67 labeling (Fig. 2).

By scoring and grouping according to low or high AHCYL1 expression, we found a mild or weak AHCYL1 expression in men and a higher expression in women (Table 1) and a statistical significance in the inverse correlation between Ki67 and AHCYL1 expression (Spearman's correlation analysis; *p* = 0.002).

**Table 1:** Clinical and histopathological parameters of surgical samples from patients with adenocarcinoma according to the expression level of AHCYL1

	Total no	AHCYL1 low no (%)	AHCYL1 high no (%)	*p* value
	20	5 (25%)	15 (75%)	
Age (years ± SD)	64.9 (9.9)	67.3 (8.2)	64.0 (10.6)	0.539^a^
Gender (%)				0.038*^b^
Male	11 (55%)	5 (100%)	6 (40%)	
Female	9 (45%)	0 (0%)	9 (60%)	–
Follow-up time (median-IQR)	452 (30–1976)	379(365–833)	1694 (1246–2535)	0.098^c^
Tumor size (mm ± SD)	29.5 (16.5)	24.2 (9.9)	31.3 (18.1)	0.417^a^
Pleural infiltration (%)	8 (40%)	2 (40%)	6 (40%)	1.000^b^
Histologic grade (3 vs. 1–2)	9 (45%)	3 (60%)	6 (40%)	0.396^b^
UICC TNM Stage (II–III)	7 (35%)	1 (20%)	6 (40%)	0.406^b^
Ki67 (% median-IQR)	15 (5–35)	60 (30–70)	5 (5–17)	0.004*c

Data are expressed as absolute numbers (%), mean (SD), or median (interquartile range)

*Corresponds to statistically significant differences (*p* < 0.05) evaluated by ^a^Student’s t-test, ^b^Fisher’s exact test, ^c^Mann-Whitney test

A survival study using Cox regression with univariate analysis considering histologic grade, age, sex, stage, Ki67, and AHCYL1 of patients revealed that Ki67 and AHCYL1 are potential predictors (Ki67, HR 95% CI 1.048 (1.007–1.090), *p* = 0.022; AHCYL1 HR (hazard ratio), 0.169 (0.033–0.869),* p* = 0.033) (Additional file 7: Table S5), suggesting AHCYL1 as a protective variable and Ki67 as a variable of greater risk.

### Downregulated *AHCYL1* expression in LCSCs

**Fig. 3:**
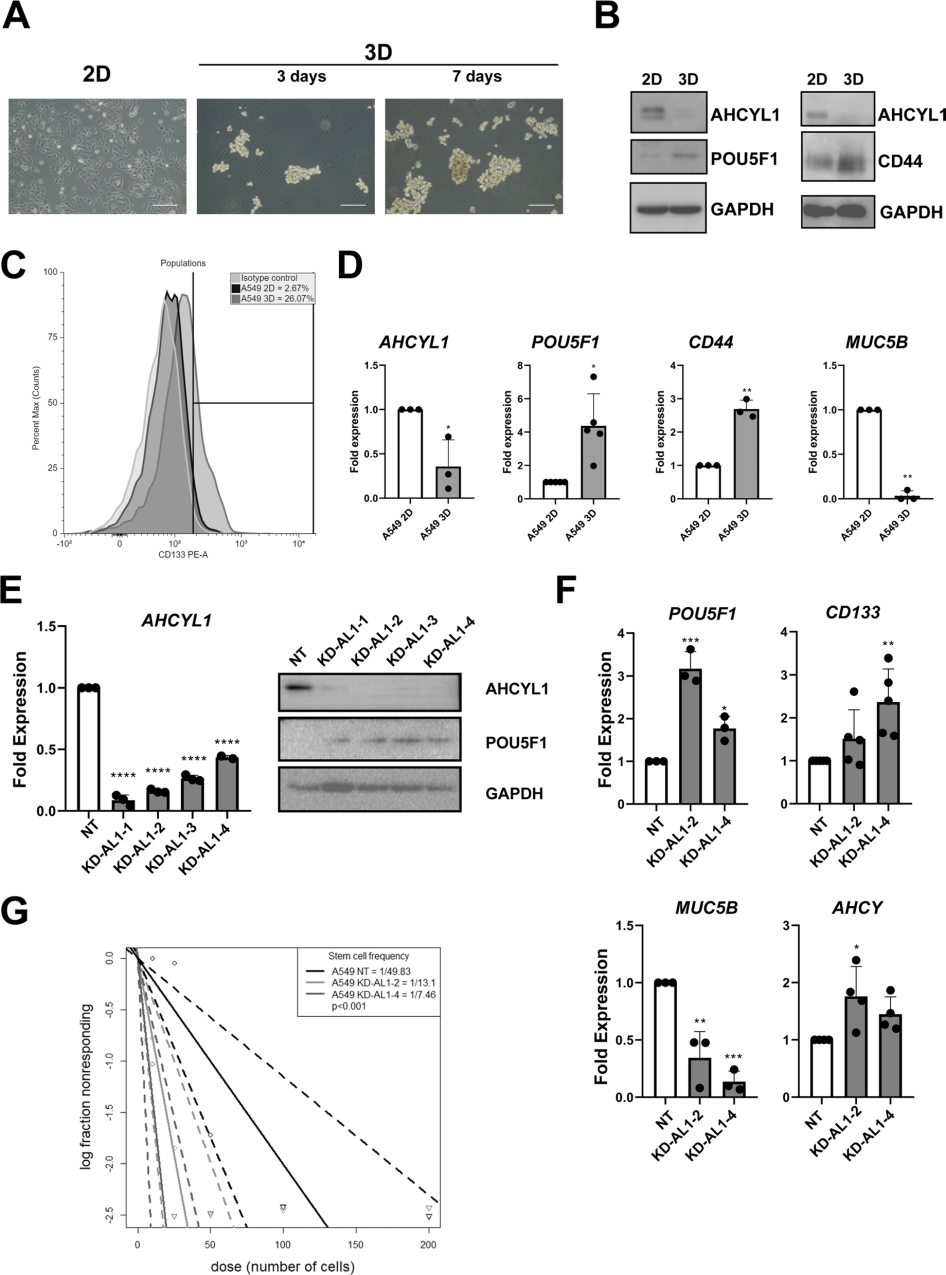
AHCYL1 expression in 3D-culture in A549 cell line and cell differentiation states of stably silenced AHCYL1 cells. **A** Representative phase-contrast microscopy images of human A549 LC cells grown as monolayers (2D) and spheroids (3D) culture enriched in LCSCs at 3 and 7 days. Ref: 1 mm. **B** Western Blot analysis of AHCYL1 (60 kDa), POU5F1 (48 kDa), and CD44 (75 kDa) protein levels of 2D and 3D culture of A549 lung adenocarcinoma cell line. GAPDH (37 kDa) was used as a loading control. The samples correspond to spheroids of 7 days. The blot corresponds to a representative experiment of three. **C** FACS analysis of CD133 expression in A549 monolayer vs spheres cultures. A549 spheres are enriched in CD133 expression. **D** qRT-PCR analysis of AHCYL1, stem cell markers *POU5F1* and *CD44* and lung marker *MUC5B*. Gene expression levels in sphere were normalized to their expression in monolayer cultures. *RPL19* was used as a normalization control. T-test with Welch's correction (n = 3–5). **E** qRT-PCR analysis of KD-AL1-1, AL1-2, AL-1-3, and AL-1-4 A549 cells lines showing significantly decreased expression of *AHCYL1* mRNA levels compared to non-targeting (NT) control cells. *RPL19* was used as a normalization control. ANOVA followed by Dunnet's test (n = 3). Western blot analysis showing AHCYL1 protein level decreased for each line and increased POU5F1 (48 kDa) protein level. GAPDH (37 kDa) was used as a loading control. The blot corresponds to a representative experiment of three. **F** RT-qPCR analyzing the expression of the pluripotency markers *POU5F1*, *AHCY*, *CD133*, and *MUC5B* as differentiation marker in the lung in KD-AL1-2 and KD-AL1-4 cells compared to NT control cells. *RPL19* was used as a normalization control. ANOVA followed by Dunnet’s test (n = 3). **G** Stem cell frequency was calculated using online Extreme Limiting Dilutions Assay (ELDA) analysis program. Significant differences in stem cell frequencies was determined between NT (1/49.83) and KD-AL1-2 (1/13.10) or KD-AL-1–4 (1/7.46) cells. The graph corresponds to a representative test (n = 3, *p* ≤ 0.001, in six replicates). The solid line shows the mean and the dotted lines show the confidence interval. *p < 0.05, **p < 0.01, ***p < 0.001, ****p < 0.0001

To further assess a potential link between AHCYL1 expression and cell identity status, AHCYL1 expression level was analyzed in A549 and H1299 cells grown in stemness conditions. The spheroid-forming cells showed consistent downregulation of *AHCYL1* expression (Fig. 3A–D, and Additional file 8: Fig. S2), along with an increased expression of the stem cell markers *POU5F1, CD44,* and *CD133* (Fig. 3B, C, and Additional file 8: Fig. S2). Accordingly, a lower expression of *Mucin 5B* (*MUC5B*) and corresponding to the lung differentiation marker [38] was observed (Fig. 3D). Altogether, we concluded that AHCYL1 expression is downregulated in undifferentiated stem-like lung cancer cells.

### *AHCYL1* expression regulates cell differentiation status in NSCLC

Next, we evaluated if AHCYL1 could regulate the stemness properties of cells in LC. To test this, stable knocked-down of AHCYL1 in human LC A549 and H1299 cell lines was performed using four independent shRNAs, targeting the coding region (referred as KD-AL1-1, -2, and 4) and 3′ UTR (KD-AL1-3) of the *AHCYL1* mRNA, respectively. A non-targeting hairpin (NT) shRNA was included in the assay as a control. Knockdown efficiency was similar with all four shRNAs (Fig. 3E and Additional file 8: Fig. S2). A significant increase of *POU5F1*, *CD133, AHCY* was observed (Fig. 3F) in KD AL1-2 and -4 representative cell lines. Similar results were observed with H1299 cells (Additional file 8: Fig. S2). In contrast, significantly decreased expression of *MUC5B* and *SFTPC* was evidenced in *AHCYL1* silenced cells (Fig. 3F and Additional file 8: Fig. S2), suggesting these cells were less differentiated than control NT treated cells.

Next, we evaluated the self-renewal capacity of *AHCYL1* silenced cells using limiting dilution assays in suspension [31]. We observed that the number of A549 KD-AL1 cells–derived spheres was significantly higher compared with NT-derived spheres, indicating more self-renewal capacity, closely associated with an increased tumor initiation capacity (Fig. 3G). The quantification of spheres/area for other A549 silenced lines (Additional file 9: Fig. S3), also confirmed that the decrease in the expression of AHCYL1 increases the ability to form tumor spheres. To validate our results, the self-renewal capacity of H1299 KD-AL1 cells was also measured, which produced similar results (Additional file 8: Fig. S2), indicating that AHCYL1 can regulate the cell differentiation status in LC cells.

AHCYL1 was previously characterized as a regulator of ribonucleotide reductase (RNR), which plays roles in cell cycle progression. Therefore, we evaluated cell proliferation rate in AHCYL1 silenced cells and found no significant differences in the proliferation rates (Additional file 10: Fig. S4). To confirm these results, FACS analysis also revealed that silencing of AHCYL1 did not modify their cell cycle progression (Additional file 10: Fig. S4). Collectively, these results indicated that the downregulation of *AHCYL1* expression does not affect their cell proliferation in vitro in LC cell lines.

### *AHCYL1* depletion increases tumorigenic capacity in vivo

**Fig. 4:**
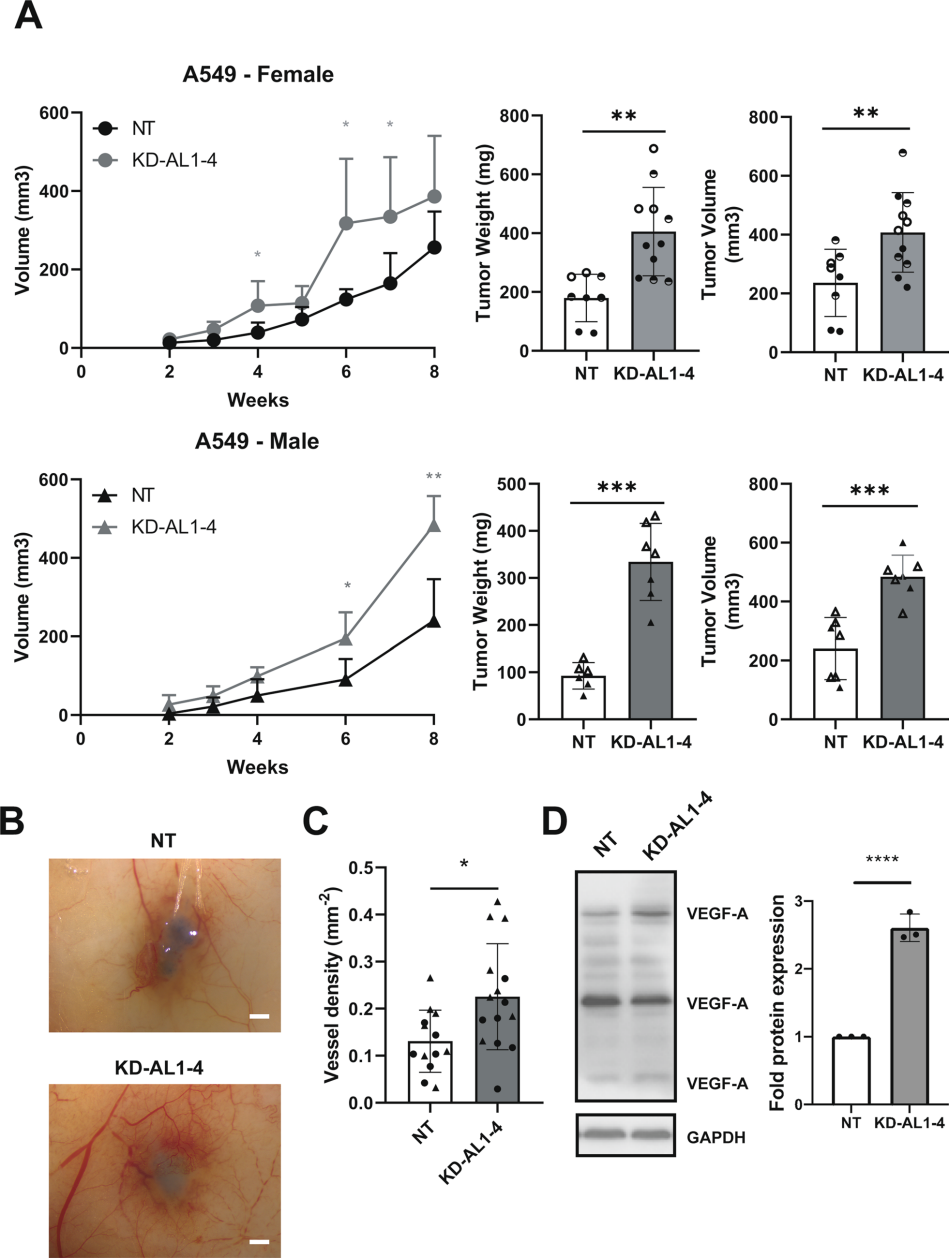
Silencing of AHCYL1 increases the tumorigenic and angiogenic capacities in vivo. **A**–**F** NOD*scid* Female mice (n = 8–11 mice per group), and Male (n = 7–8 mice per group) were subcutaneously injected with 2 × 10^6^ A549 cells stably expressing control shRNA (NT) or AHCYL1 shRNA (KD-AL1-4). Average tumor volume ± SD is plotted against time (in days). Different shading corresponds to two independent experiments. Differences were evaluated using a Repeated Measures Design. Final tumor weight and volume are also shown. Means were compared using ANOVA followed by Dunnet’s test. **B** A549 cells (10^6^, cells, each condition) were intradermal injected in the right flank of NOD*scid* male mice (n = 6–7 mice per group). Vehicle (DMEM without FBS) was injected in the left flank. After 7 days, photographs of skin were taken under magnification glass to quantify vessel density. Representative photograph for each condition (NT vs KD-AL1-4) are shown. Bar: 5 mm. **C** Quantification (mm^2^) of vessel density in each condition (n = 13–15 per group). Circles and triangles are used to identify individuals from two independent experiments. Means were compared using a T-test. **D** Western Blot of VEGF-A (23, 27 and 42 kDa) in NT control and AHCYL1-depleted cells (KD-AL1-4). Quantification of VEGF band (23 kDa) from the western blot of **D** showing decreased VEGF protein level in AHCYL1 silenced cells (KD-AL1-4). *p < 0.05, **p < 0.01, ***p < 0.001

To gain further insight into the pathophysiological role of AHCYL1 in NSCLC cells, we evaluated the impact of AHCYL1 downregulation during tumor development. AHCYL1 silenced cells were subcutaneously injected into the flank of NOD*scid* mice and tumor size and weight were monitored (Fig. 4A and Additional file 11: Fig. S5). A549 KD-AL1-4 derived tumors were significantly larger compared to NT controls (Fig. 4A), although the observable effect was stronger in males (Fig. 4A). Silencing of AHCYL1 also increased in vivo tumor capacity in H1299 cells (Additional file 11: Fig. S5). These results indicated a potential role of AHCYL1 as a tumor suppressor in lung cancer.

### Down regulation of *AHCYL1* expression enhances tumor angiogenic capacity in vivo

Angiogenesis is a critical process driving cancer progression and plasticity and is associated with poor prognosis in LC [39, 40]. Therefore, we evaluated the angiogenic capacity of A549 KD-AL1-4 cells (see Additional file 5) [41]. After 7 days, tumor vessel densities were quantified visually (Fig. 4B, C) [42]. Vessel density was significantly higher around the injection area of KD-AL1-4 tumors (Fig. 4C). Accordingly, we also observed that AHCYL1-depleted cells showed increased VEGF-A protein levels, compared to NT-control cells (Fig. 4D). Thus, our results suggest that AHCYL1 expression affects angiogenesis in NSCLC.

### Down regulation of *AHCYL1* expression affects metabolic balance in LC cells

**Fig. 5:**
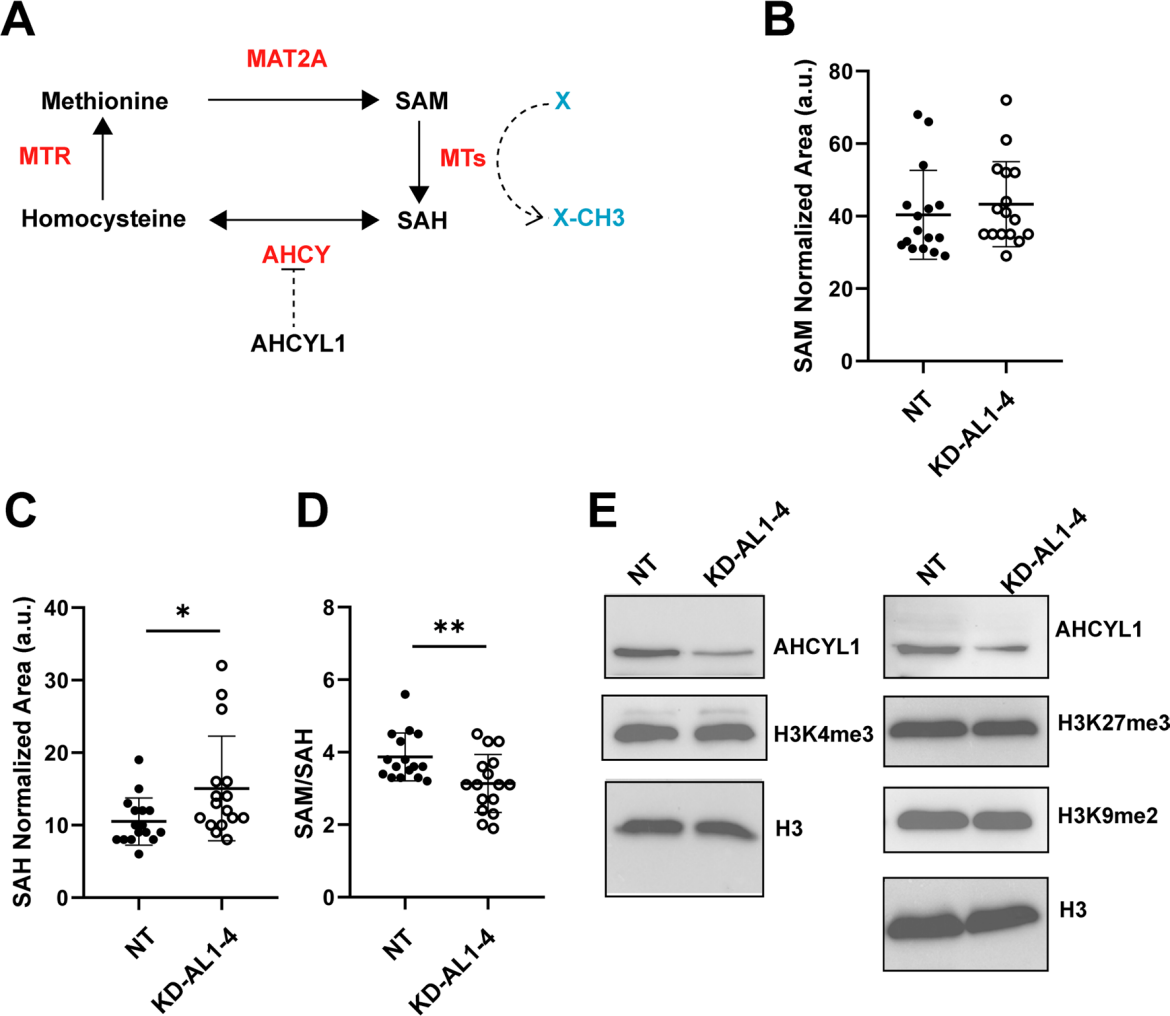
Detection of SAM and SAH by UPLC-QTOF-MS. **A** Schematic representation of the methionine cycle, the proposed inhibitory action of AHCYL1 on AHCY, and the interaction with SAH. MTR: Methionine synthase. MAT2A: Methionine adenosyl transferase 2A. Normalized chromatographic peak areas for SAM (**B**) and SAH (**C**) for NT and KD-AHCYL1-4 cells (n = 16 for each cell line,* p* = 0.026). **D** SAM/SAH ratio for NT and KD-AHCYL1-4 cells calculated for each sample (n = 16, *p* = 0.007). *p ≤ 0.05, **p ≤ 0.01. **E** Western blot analysis of AHCYL1 protein level and H3K4,-K9 and -27 methylation levels. Total histone H3 was used as a loading control. The blot corresponds to a representative experiment of two or three blots with similar results

AHCY is an enzyme responsible for the reversible conversion of SAH during the methionine cycle (Fig. 5A) [10], highly expressed in LC cells [22], and directly interacts with AHCYL1 [17, 19]. We evaluated whether AHCYL1 expression has an impact on the methionine cycle, therefore, intracellular levels of SAM and SAH metabolites were determined in KD-AL1-4 cells by means of UPLC-QTOF-MS. No significant difference in SAM levels was observed between control NT and KD-AL1-4 (Fig. 5B), however, SAH levels were slightly but significantly elevated in KD-AL1-4 (Fig. 5C).

The SAM/SAH ratio is attributed to determine the methylation potential in cells [43]. In KD-AL1-4, the SAM/SAH ratio values were significantly lower (Fig. 5D), although with little differences, suggesting a minor reduced methylation potential, in apparent contradiction with the higher tumorigenic capacity observed (Fig. 4). The trimethylation of lysine 4 of histone 3 (H3K4me3) levels, as well as methylation status of H3K9 and H3K27, are considered to be correlated with methylation potential, showed no significant difference in KD AL1-4 cells (Fig. 5E) [22]. Altogether, these results suggest that the small reduction of SAM/SAH values triggered by AHCYL1 silencing does not affect the methylation potential in KD-AL1-4 cells.

## Discussion

We found that *AHCYL1* mRNA expression is reduced in the tumor cell population that co-expressed stemness genes, likewise in LUAD patient samples with recurrence, suggesting *AHCYL1* expression could be associated with a more differentiated phenotype in lung samples. Notably, AHCYL1 protein level was also lower regardless of the cancer grade, and the decreased expression of AHCYL1 was more evident in more aggressive tumors, being inversely correlated with the expression of the tumor marker Ki67, which is widely used to determine the degree of growth, invasion, and prognosis in cancer [35, 36].

Accordingly, the transcriptomic data showed that patients with low *AHCYL1* and high *Ki67* expression exhibited worse prognosis. Our univariate statistic test using 20 cases indicated that the expression of AHCYL1 and Ki67 determined by IHC could be explored as potential indicator of survival. Based on these results, we concluded a protective role for AHCYL1 and Ki67 as an indicator of greater risk. Therefore, we consider that by increasing the number of biopsies, a multicenter study using different antibodies, dilutions, detection, and amplification systems, would ascertain the use of AHCYL1 expression as a biomarker in LC in the future. In support of this notion, it was reported that epithelial ovarian cancer patients expressing intermediate to high levels of AHCYL1 showed a greater response to treatments and highest survival rate [44]. Remarkably, we also observed a significant association between AHCYL1 levels and gender, being highly expressed in all female samples compared to male. The incidence and mortality rates are twofold higher in men than in women. Notably, the tumorigenic effects of lung adenocarcinoma AHCYL1 silenced cells were stronger in male mice, highlighting the potential gender-dependency of AHCYL1 role as a tumor suppressor.

The transcriptomic data also revealed that LC tumors with lower expression of *AHCYL1* have higher pluripotency score expression. Furthermore, silencing of AHCYL1 in NSCLC cell lines and LCSC-enriched spheres allowed us to determine that AHCYL1 plays a role in tumorigenesis by hampering their self-renewal capacity in vitro and tumor growth in vivo. Moreover, our results suggest that AHCYL1 expression affects angiogenesis in NSCLC.

The activation of the methylation cycle (1C) occurs during cell reprogramming, probably associated with the establishment and maintenance of a stem cell-like state [22, 23, 45]. In support of this notion, increased AHCY expression, and elevated H3K4me3 were reported in multiple cancer types with poor prognostic, including non‐small cell lung cancer [22, 23, 46, 47]. In this regard, we found that AHCYL1-depleted cells showed a slight increase of intracellular SAH, with no change in SAM levels, decreasing the SAM/SAH values, although to a small degree, and suggesting a lower methylation capacity in these cells. However, AHCYL1-silenced cells did not display changes in the H3K4me3, H3K9me2, and H3K27me3 marks, although showed an increase in the expression of AHCY along with other stemness markers and stem cell like phenotype. These results suggested that the AHCYL1 levels affect the metabolic status of LC cells, but to a minor degree, and seems not to be associated with changes of histone methylation status. Therefore, we propose the stem cell like phenotype observed in AHCYL1 depleted LC cells is not necessarily linked to methylation potential, as described previously [22].

Overall, we have provided experimental evidence supporting a role for AHCYL1 as an inhibitor of the stem-like signature in LC and negative regulator of tumorigenesis. Further research will be required to elucidate the mechanisms by which AHCYL1 modulates stemness and its potential application as a prognostic biomarker alone or in combination with other biomarkers.

## Conclusions

We present a first evidence linking S-adenosylhomocysteine hydrolase-like protein 1 (AHCYL1) as a novel negative regulator of Non-Small Cell Lung Cancer. We determined that AHCYL1 regulates tumorigenesis by modulating the cell stemness, thus, highlighting it as a potential prognostic biomarker for lung cancer.

## Supplementary Information

**Additional file 1**. **Table S1**. Antibodies.

**Additional file 2**. **Table S2**. Primers sequences.

**Additional file 3**. **Table S3**. AHCYL1 shRNA target sequences, target region and construct number.

**Additional file 4**. **Fig. S1**: AHCYL1 INSECT analysis: SOX2/POU5F1 binding sites. SOX2/OCT4 cis-regulatory module (CRM) in silico search performed over the human AHCYL1 gene (Ensembl ID ENSG00000168710) using the INSECT 2.0 tool. OCT4 (POU5F1) was selected as the master transcriptional factor of the CRM having a SOX2 binding site in the same orientation at a maximum distance of 4 bp. The search of the motifs was performed by using the Position Weight Matrix (PWM) referred to the Swiss Regulon for POU5F1 p2 (MAT1816) and SOX2p2 (MAT2068).

**Additional file 5**. Extended Protocols.

**Additional file 6**. **Table S4**. Clinical and histopathological data from patients with lung cancer.

**Additional file 7**. **Table S5**. Univariate Cox regression to compare survival in patients with lung adenocarcinoma.

**Additional file 8**. **Fig. S2**: AHCYL1 expression in 3D-culture in NSCL H1299 cell line and cell differentiation states of stably AHCYL1-silenced H1299 and A549 cells. (A) Representative phase-contrast microscopy images of human H1299 LC cells grown as monolayers (2D) and spheroids (3D) culture enriched in LCSCs at 3 and 7 days. Ref: 1 mm. (B) qRT-PCR analysis of AHCYL1, stem cell markers (i.e. POU5F1 and CD44) and lung marker MUC5B. Gene expression levels in sphere were normalized to their expression in monolayer cultures. RPL19 was used as a normalization control. T-test with Welch's correction (n=3). (C) Western Blot analysis of AHCYL1 (60 kDa) and POU5F1 (48 kDa) protein levels of 2D and 3D culture of H1299 lung carcinoma cell line. GAPDH (37 kDa) was used as a loading control. The samples correspond to spheroids of 7 days. The blot corresponds to a representative experiment of three. (D) qRT-PCR analysis of KD-AL1-1, AL1-2, AL-1-3, and AL-1-4L H1299 cells lines showing decreased expression of AHCYL1 mRNA levels compared to non-targeting (NT) control cells. RPL19 was used as a normalization control. ANOVA followed by Dunnet's test (n=3). (E) Western blot analysis showing AHCYL1 protein level decreased for each line and POU5F1 (48 kDa) protein level increased. GAPDH (37 kDa) was used as a loading control. The blot corresponds to a representative experiment of three. (E) RT-qPCR analyzing the expression of the pluripotency markers (i.e POU5F1, AHCY, and CD133) and MUC5B as lung marker in KD-AL1-2 and KD-AL1-4 cells compared to NT control cells. RPL19 was used as a normalization control. ANOVA followed by Dunnet's test (n=3). (F) Stem cell frequency was calculated using online Extreme Limiting Dilutions Assay (ELDA) analysis program. Significant differences in stem cell frequencies was determined between NT (1/58.85) and KD-AL1-2 (1/15.33) or KD-AL-1-4 (1/16.42) cells. The graph corresponds to a representative test (n=3, p=0.002, in six replicates). The solid line shows the mean and the dotted lines show the confidence interval. (G) RT-qPCR analyzing the expression of the pluripotency markers NANOG and SFTPC as lung marker in A549 KD-AL1-2 and KD-AL1-4 cells compared to NT control cells. RPL19 was used as a normalization control. ANOVA followed by Dunnet's test (n=3). *p<0.05, **p<0.01 ****p<0.0001.

**Additional file 9**. **Fig. S3**: AHCYL1 modulates self-renewal capacity of LC cells. (A) Phase-contrast microscopy images of NT and AHCYL1 knockdown KD-AL1-1, AL1-2, AL-1-3, and AL-1-4L A549 spheres. Photographs taken of the silenced lines to quantify the percentage of area covered. Scale bar: 500 µm. (B) Percentage of the area occupied by spheres. The relative area is about the area occupied by the NT control spheres. The different symbols correspond to independent experiments (n = 2, in quadruplicate). Analyzed by Kruskal-Wallis followed by Dunn's test.

**Additional file 10**. **Fig. S4**: AHCYL1 depletion did not affect LC cells proliferation. Resazurin based proliferation assay at 48 h of A549 cells expressing NT, KD-AL1-2 or KD-AL1-4 vectors and comparison of means by ANOVA, (n=3). Crystal violet based proliferation assay at 48 h of A549 cells expressing NT, KD-AL1-2 or KD-AL1-4 vectors comparison of means by ANOVA with Brown-Forsythe and Welch correction (n=5). Doubling time of each A549 cells expressing NT, KD-AL1-2 or KD-AL1-4 vectors estimated from crystal violet method based time curve (n=5). Resazurin based proliferation assay at 48 h of H1299 cells expressing NT, KD-AL1-2 or KD-AL1-4 vectors and comparison of means by ANOVA, n=3). FACS analysis of AHCYL1 silencing in A549 cells did not modify their cell cycle progression. Cell cycle assay on A549-silenced lines performed with propidium iodide. The percentages of cells in each phase of the cycle were plotted. The values shown correspond to technical quintuplicate (n=1). Means were compared using ANOVA and no significant differences were observed.

**Additional file 11**. **Fig. S5**: Female NODscid mice subcutaneously injected with H1299 KD-AL1-4 cells. (A) Average tumor volume ± SD is plotted against time (in days). Differences were evaluated using a Repeated Measures Design. (B) Final Tumor Volume. Means were compared using ANOVA followed by Dunnet's test. (C) Final weight of the tumors. Means were compared using ANOVA followed by Dunnet's test. *p≤0.05.

## Data Availability

The datasets and material generated during the current study are available upon request to cperezcastro@ibioba-mpsp-conicet.gov.ar.
