# Correction: S-adenosylhomocysteine hydrolase-like protein 1 (AHCYL1) inhibits lung cancer tumorigenesis by regulating cell plasticity

**DOI:** 10.1186/s13062-023-00367-9

**Published:** 2023-03-29

**Authors:** Melina Muñoz-Bernart, Nicolás Budnik, Araceli Castro, Malena Manzi, María Eugenia Monge, Julieta Pioli, Sebastián Defranchi, Gustavo Parrilla, Juan Pablo Santilli, Kevin Davies, Joaquín M. Espinosa, Ken Kobayashi, Carlos Vigliano, Carolina Perez-Castro

**Affiliations:** 1 Instituto de Investigación en Biomedicina de Buenos Aires (IBioBA) - CONICET, Partner Institute of the Max Planck Society, Buenos Aires, Argentina; 2 Instituto de Medicina Traslacional, Trasplante y Bioingeniería (IMeTTyB), Universidad Favaloro-CONICET, Solís 453, C1078AAI Buenos Aires, Argentina; 3 Centro de Investigaciones en Bionanociencias (CIBION), Consejo Nacional de Investigaciones Científicas y Técnicas (CONICET), Godoy Cruz 2390, C1425FQD Ciudad de Buenos Aires, Argentina; 4 Departamento de Fisiología, Biología Molecular y Celular, Facultad de Ciencias Exactas y Naturales, Universidad de Buenos Aires, Intendente Güiraldes, 2160 C1428EGA Buenos Aires, Argentina; 5 Consejo Nacional de Investigaciones Científicas y Técnicas (CONICET), Departamento de Desarrollo Analítico y Control de Procesos, Instituto Nacional de Tecnología Industrial, Av. General Paz 5445, B1650WAB Buenos Aires, Argentina; 6 Servicio de Cirugía Torácica, Hospital Universitario de la Fundación Favaloro, Av. Belgrano 1746, C1093AAS Buenos Aires, Argentina; 7 Servicio de Anatomía Patológica, Hospital Universitario de la Fundación Favaloro, Av. Belgrano 1746, C1093AAS Buenos Aires, Argentina; 8 Linda Crnic Institute for Down Syndrome, University of Colorado Anschutz Medical Campus, Aurora, CO USA; 9 Department of Pharmacology, University of Colorado Anschutz Medical Campus, Aurora, CO USA; 10 Department of Molecular, Cellular and Developmental Biology, University of Colorado Boulder, Boulder, CO USA; 11 Laboratorio de Agrobiotecnología, Instituto de Biodiversidad y Biología Experimental Aplicada (IBBEA-CONICET-UBA), Facultad de Ciencias Exactas y Naturales, Universidad de Buenos Aires, Buenos Aires, Argentina

**Correction to: Biology Direct (2023) 18:8**

10.1186/s13062-023-00364-y

After publication of this article [1], it was brought to our attention that one of the second author’s name is misspelled, it should be changed from Nicolás Budnick to Nicolás Budnik.

The original publication has been corrected.
