# Supplementary material for: S-adenosylhomocysteine hydrolase-like protein 1 (AHCYL1) inhibits lung cancer tumorigenesis by regulating cell plasticity
Source: Biol Direct. 2023 Mar 5;18:8. doi: 10.1186/s13062-023-00364-y (PMC9985837; doi:10.1186/s13062-023-00364-y)
Supplement: Supplementary file 1 — Additional file 1. Table S1. Antibodies. [file 13062_2023_364_MOESM1_ESM.docx]

Supplementary Table 1. Antibodies

| **Antibody Target** | **Catalog** | **Research Resource Identifiers (RRID)** | **Supplier** |
| --- | --- | --- | --- |
| AHCYL1 | SAB21000076 | RRID:AB_10599407 | Sigma-Aldrich |
| Ki67 | #790-4286 | RRID:AB_2631262 | Ventana Medical Systems |
| POU5F1 | ab19857 | RRID:AB_445175 | Abcam |
| H3K4me3 | ab8580 | RRID:AB_306649 | Abcam |
| H3 | 4499 | RRID:AB_10544537 | Cell Signaling |
| VEGF | 19003-1-AP | RRID:AB_2212657 | Proteintech |
| CD44 | sc 7297 | RRID:AB_627065 | Santa Cruz |
| H3K9me2 | 4658 | RRID:AB_10544405 | Cell Signaling |
| H3K27me3 | ab6002 | RRID:AB_305237 | Abcam |
| CD133 | AC133-PE | RRID:AB_244342 | Miltenyi Biotec |
| GAPDH | ab8245 | RRID:AB_2107448 | Abcam |
