# Supplementary material for: S-adenosylhomocysteine hydrolase-like protein 1 (AHCYL1) inhibits lung cancer tumorigenesis by regulating cell plasticity
Source: Biol Direct. 2023 Mar 5;18:8. doi: 10.1186/s13062-023-00364-y (PMC9985837; doi:10.1186/s13062-023-00364-y)
Supplement: Supplementary file 2 — Additional file 2. Table S2. Primers sequences. [file 13062_2023_364_MOESM2_ESM.docx]

**Supplementary Table 2. Primers sequences.**

| **Primer** | **Sequence (5’-3’)** |
| --- | --- |
| Human AHCYL1 Forward | CGATGTGACCAGCCTCCGCAC |
| Human AHCYL1 Reverse | TGCTTGTATCGCCCCTCGGGT |
| Human AHCY Forward | ATCCTTGGCCGGCACTTTGAG |
| Human AHCY Reverse | TCCACCTGCGGCTTGATGTTC |
| Human CD44 Forward | TCCAACACCTCCCAGTATGACA |
| Human CD44 Reverse | GGCAGGTCTGTGACTGATGTACA |
| Human CD133 Forward | ACAACACTACCAAGGACAAGG |
| Human CD133 Reverse | GGACTTAATCTCATCAAGAACAGG |
| Human MUC5B Forward | TACAACGTGCGTGTGCTTTG |
| Human MUC5B Reverse | CTGTTGTGGTCGGCTTTGTG |
| Human POU5F1 Forward | GCTGGAGAAGGAGAAGCTGG |
| Human POU5F1 Reverse | GCTAAGCTGCAGAGCCTCAA |
| Human RPL19 Forward | CAATGCCAACTCCCGTCAGCAGAT |
| Human RPL19 Reverse | GTGTTTTTCCGGCATCGAGCCC |
