# Supplementary material for: S-adenosylhomocysteine hydrolase-like protein 1 (AHCYL1) inhibits lung cancer tumorigenesis by regulating cell plasticity
Source: Biol Direct. 2023 Mar 5;18:8. doi: 10.1186/s13062-023-00364-y (PMC9985837; doi:10.1186/s13062-023-00364-y)
Supplement: Supplementary file 3 — Additional file 3. Table S3. AHCYL1 shRNA target sequences, target region and construct number. [file 13062_2023_364_MOESM3_ESM.docx]

Supplementary Table 3. AHCYL1 shRNA target sequences, target region and construct number.

| **ID** | **Target Sequence** | **Target Region** | **Construct** |
| --- | --- | --- | --- |
| KD-AL1-1 | GCACTGATAGAACTCTATAAT | CDS | TRCN0000299611 |
| KD-AL1-2 | CGGCAAGTCGATGTCGTAATA | CDS | TRCN0000299610 |
| KD-AL1-3 | CAATGTCTAAATCGCCTTAAA | 3´UTR | TRCN0000303728 |
| KD-AL1-4 | GATGTGATGTTTGGTGGGAAA | CDS | TRCN0000310439 |
