# Supplementary figures and images for: S-adenosylhomocysteine hydrolase-like protein 1 (AHCYL1) inhibits lung cancer tumorigenesis by regulating cell plasticity
Source: Biol Direct. 2023 Mar 5;18:8. doi: 10.1186/s13062-023-00364-y (PMC9985837; doi:10.1186/s13062-023-00364-y)

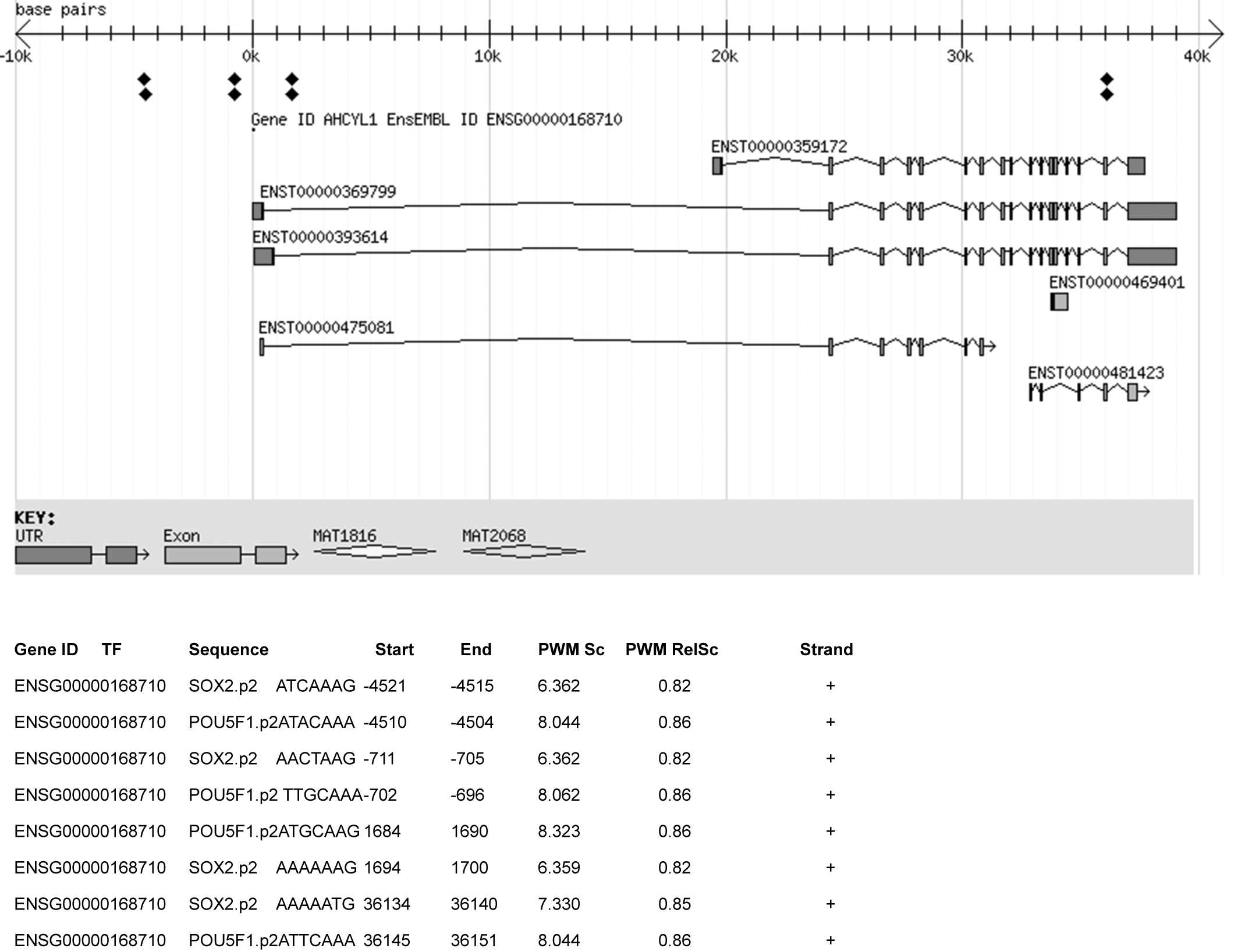

Supplement: Supplementary file 4 — Additional file 4. Fig. S1: AHCYL1 INSECT analysis: SOX2/POU5F1 binding sites. SOX2/OCT4 cis-regulatory module (CRM) in silico search performed over the human AHCYL1 gene (Ensembl ID ENSG00000168710) using the INSECT 2.0 tool. OCT4 (POU5F1) was selected as the master transcriptional factor of the CRM having a SOX2 binding site in the same orientation at a maximum distance of 4 bp. The search of the motifs was performed by using the Position Weight Matrix (PWM) referred to the Swiss Regulon for POU5F1 p2 (MAT1816) and SOX2p2 (MAT2068). [file 13062_2023_364_MOESM4_ESM.tif]

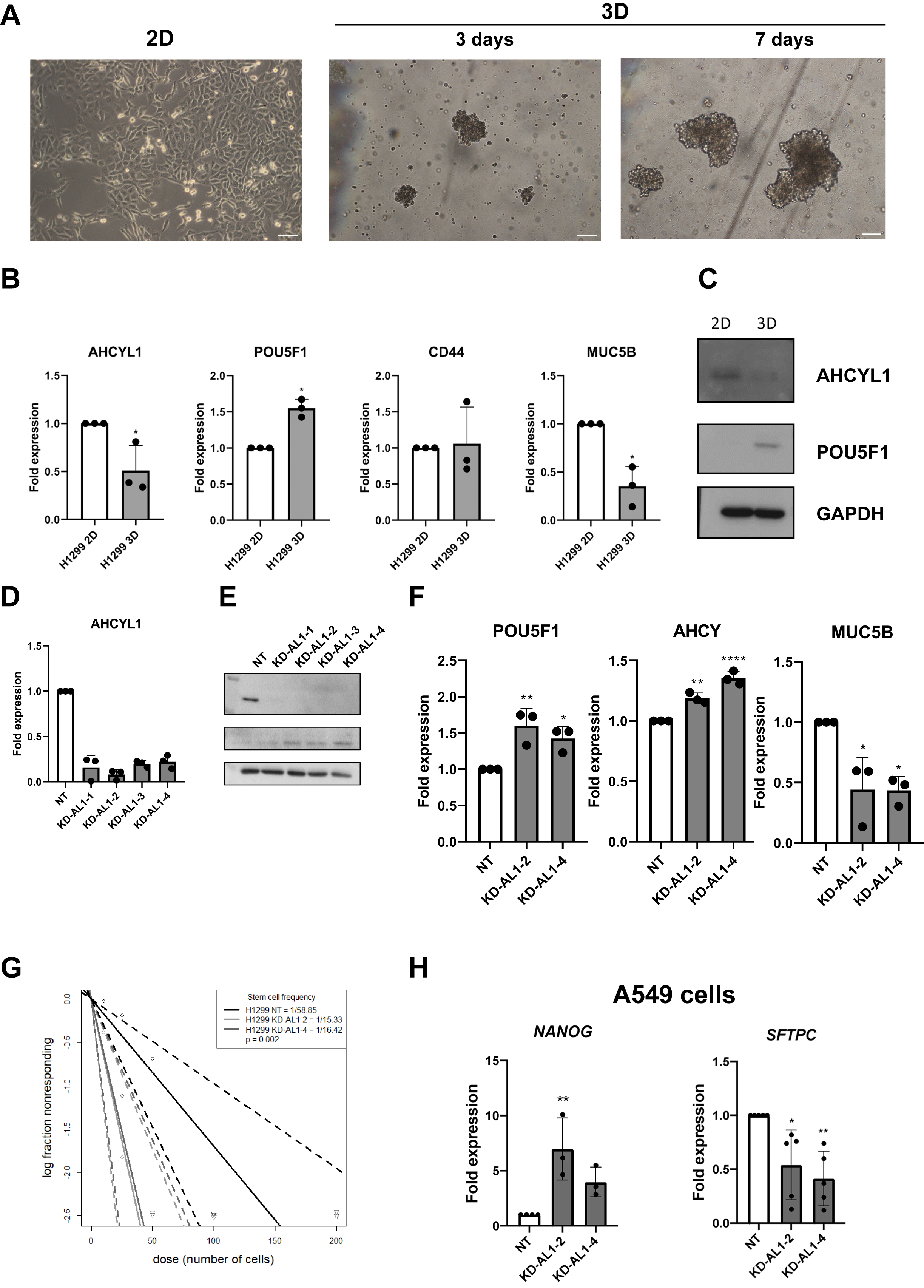

Supplement: Supplementary file 8 — Additional file 8. Fig. S2: AHCYL1 expression in 3D-culture in NSCL H1299 cell line and cell differentiation states of stably AHCYL1-silenced H1299 and A549 cells. (A) Representative phase-contrast microscopy images of human H1299 LC cells grown as monolayers (2D) and spheroids (3D) culture enriched in LCSCs at 3 and 7 days. Ref: 1 mm. (B) qRT-PCR analysis of AHCYL1, stem cell markers (i.e. POU5F1 and CD44) and lung marker MUC5B. Gene expression levels in sphere were normalized to their expression in monolayer cultures. RPL19 was used as a normalization control. T-test with Welch's correction (n=3). (C) Western Blot analysis of AHCYL1 (60 kDa) and POU5F1 (48 kDa) protein levels of 2D and 3D culture of H1299 lung carcinoma cell line. GAPDH (37 kDa) was used as a loading control. The samples correspond to spheroids of 7 days. The blot corresponds to a representative experiment of three. (D) qRT-PCR analysis of KD-AL1-1, AL1-2, AL-1-3, and AL-1-4L H1299 cells lines showing decreased expression of AHCYL1 mRNA levels compared to non-targeting (NT) control cells. RPL19 was used as a normalization control. ANOVA followed by Dunnet's test (n=3). (E) Western blot analysis showing AHCYL1 protein level decreased for each line and POU5F1 (48 kDa) protein level increased. GAPDH (37 kDa) was used as a loading control. The blot corresponds to a representative experiment of three. (E) RT-qPCR analyzing the expression of the pluripotency markers (i.e POU5F1, AHCY, and CD133) and MUC5B as lung marker in KD-AL1-2 and KD-AL1-4 cells compared to NT control cells. RPL19 was used as a normalization control. ANOVA followed by Dunnet's test (n=3). (F) Stem cell frequency was calculated using online Extreme Limiting Dilutions Assay (ELDA) analysis program. Significant differences in stem cell frequencies was determined between NT (1/58.85) and KD-AL1-2 (1/15.33) or KD-AL-1-4 (1/16.42) cells. The graph corresponds to a representative test (n=3, p=0.002, in six replicates). The solid line show [file 13062_2023_364_MOESM8_ESM.tif]

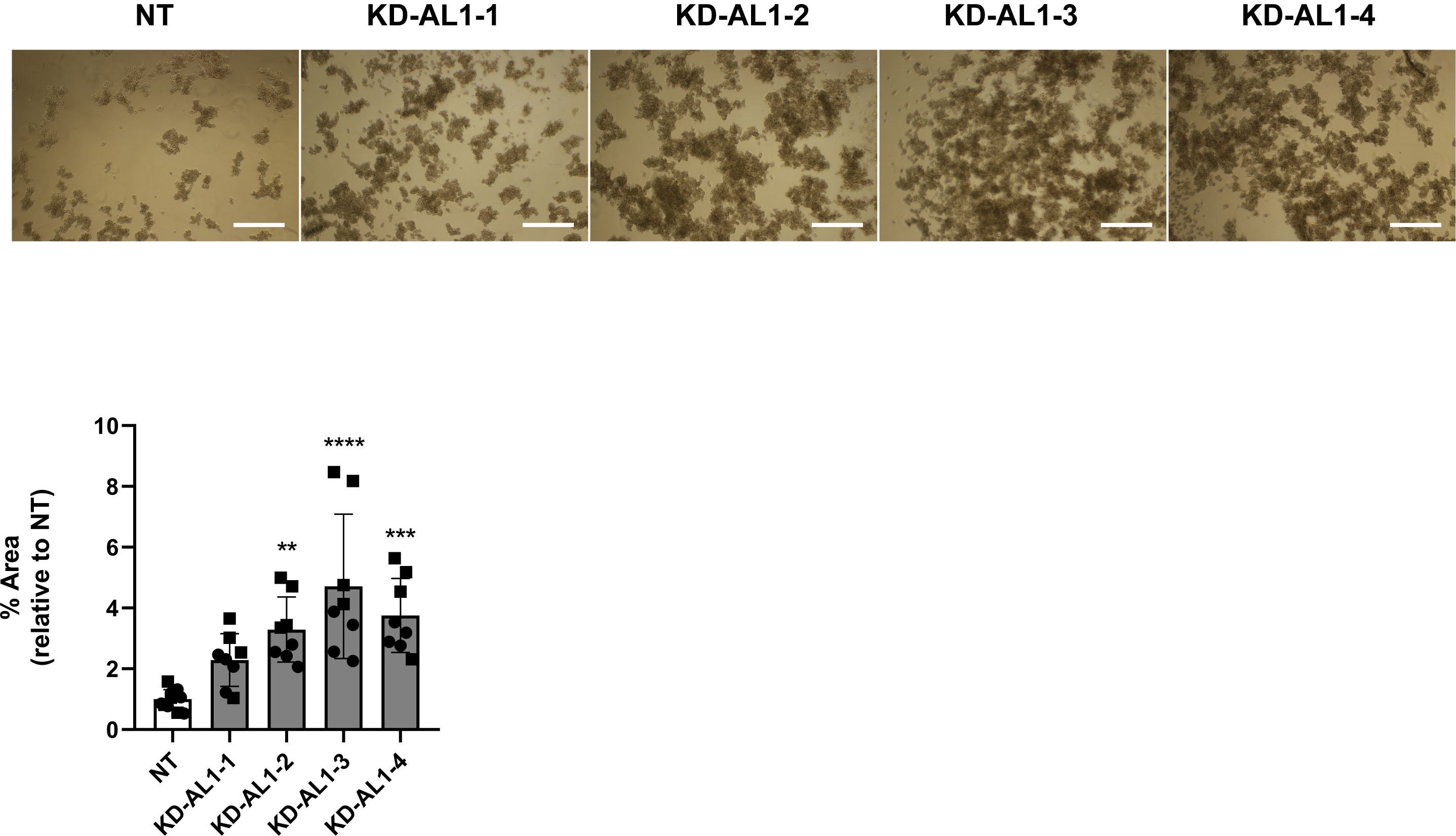

Supplement: Supplementary file 9 — Additional file 9. Fig. S3: AHCYL1 modulates self-renewal capacity of LC cells. (A) Phase-contrast microscopy images of NT and AHCYL1 knockdown KD-AL1-1, AL1-2, AL-1-3, and AL-1-4L A549 spheres. Photographs taken of the silenced lines to quantify the percentage of area covered. Scale bar: 500 µm. (B) Percentage of the area occupied by spheres. The relative area is about the area occupied by the NT control spheres. The different symbols correspond to independent experiments (n = 2, in quadruplicate). Analyzed by Kruskal-Wallis followed by Dunn's test. [file 13062_2023_364_MOESM9_ESM.tif]

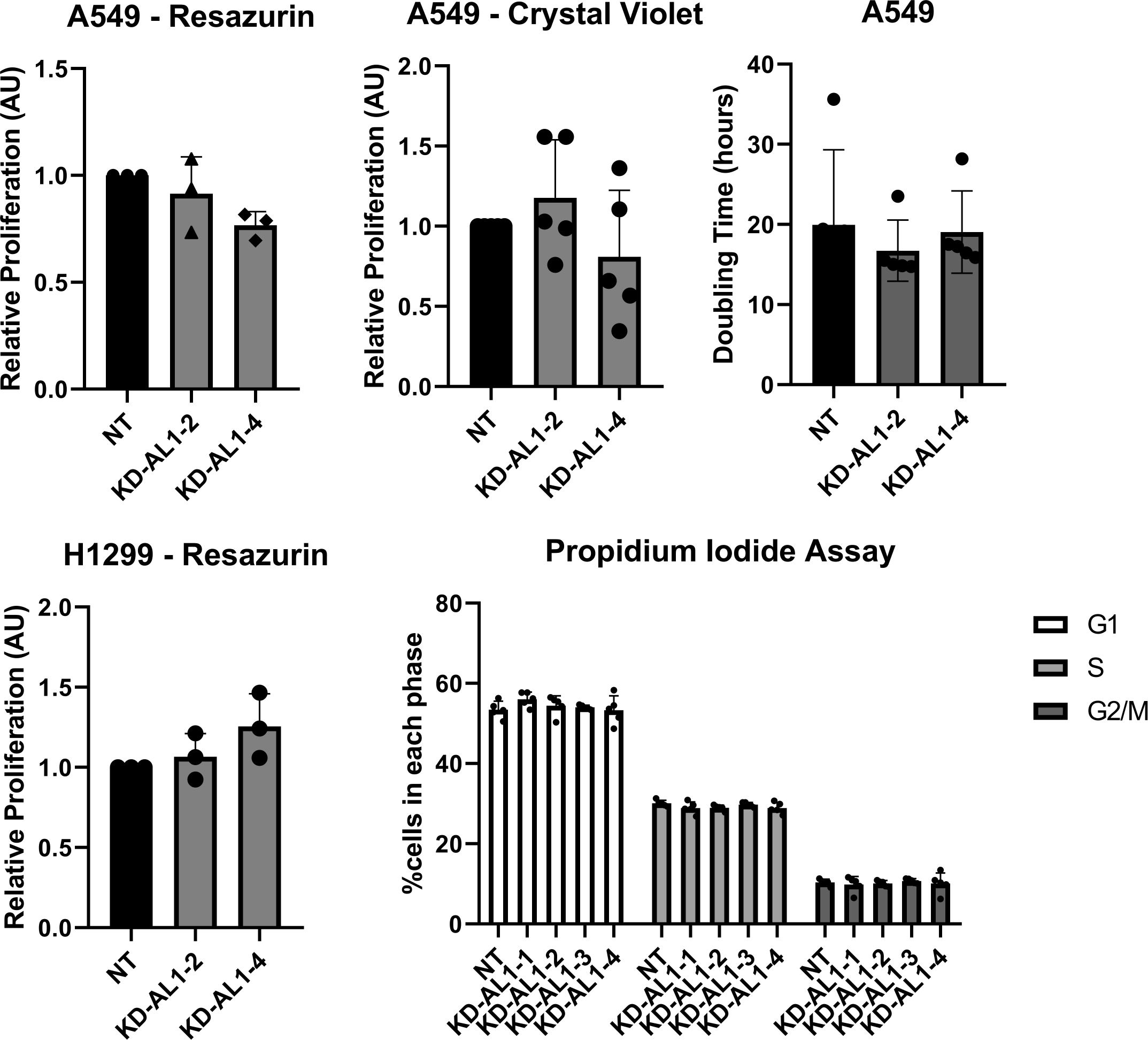

Supplement: Supplementary file 10 — Additional file 10. Fig. S4: AHCYL1 depletion did not affect LC cells proliferation. Resazurin based proliferation assay at 48 h of A549 cells expressing NT, KD-AL1-2 or KD-AL1-4 vectors and comparison of means by ANOVA, (n=3). Crystal violet based proliferation assay at 48 h of A549 cells expressing NT, KD-AL1-2 or KD-AL1-4 vectors comparison of means by ANOVA with Brown-Forsythe and Welch correction (n=5). Doubling time of each A549 cells expressing NT, KD-AL1-2 or KD-AL1-4 vectors estimated from crystal violet method based time curve (n=5). Resazurin based proliferation assay at 48 h of H1299 cells expressing NT, KD-AL1-2 or KD-AL1-4 vectors and comparison of means by ANOVA, n=3). FACS analysis of AHCYL1 silencing in A549 cells did not modify their cell cycle progression. Cell cycle assay on A549-silenced lines performed with propidium iodide. The percentages of cells in each phase of the cycle were plotted. The values shown correspond to technical quintuplicate (n=1). Means were compared using ANOVA and no significant differences were observed. [file 13062_2023_364_MOESM10_ESM.tif]

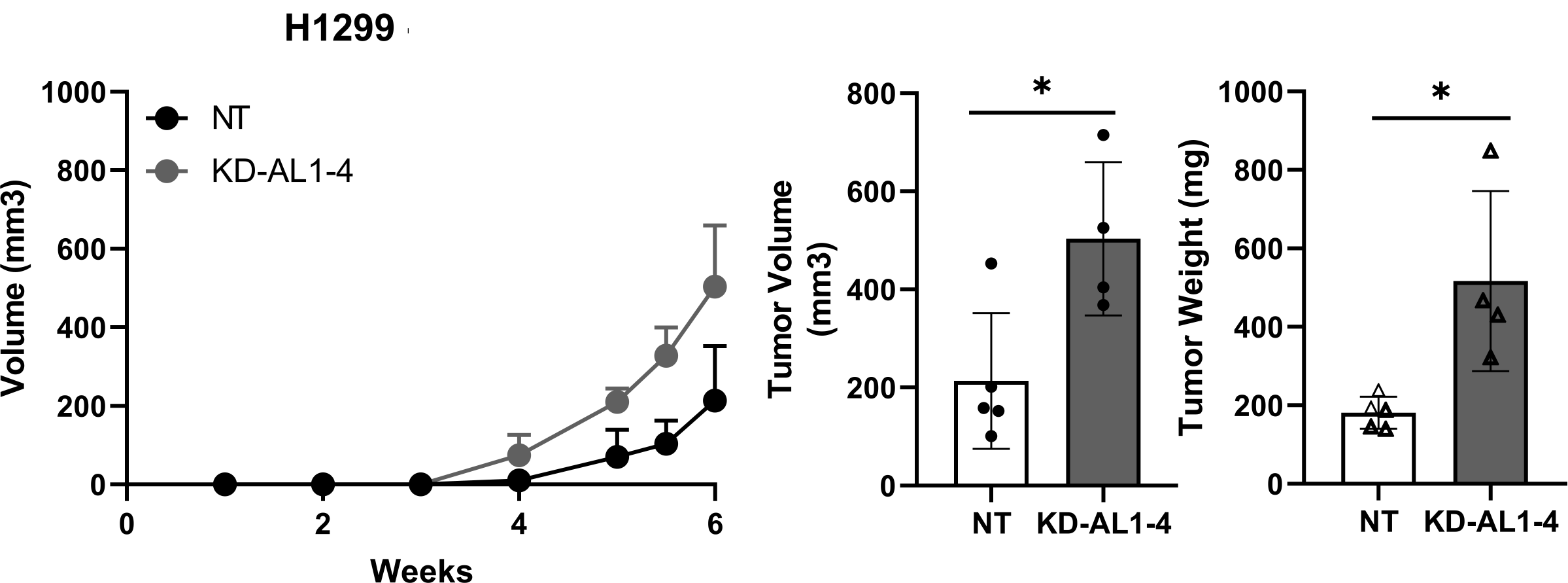

Supplement: Supplementary file 11 — Additional file 11. Fig. S5: Female NODscid mice subcutaneously injected with H1299 KD-AL1-4 cells. (A) Average tumor volume ± SD is plotted against time (in days). Differences were evaluated using a Repeated Measures Design. (B) Final Tumor Volume. Means were compared using ANOVA followed by Dunnet's test. (C) Final weight of the tumors. Means were compared using ANOVA followed by Dunnet's test. *p≤0.05. [file 13062_2023_364_MOESM11_ESM.tif]
