# Supplementary material for: S-adenosylhomocysteine hydrolase-like protein 1 (AHCYL1) inhibits lung cancer tumorigenesis by regulating cell plasticity
Source: Biol Direct. 2023 Mar 5;18:8. doi: 10.1186/s13062-023-00364-y (PMC9985837; doi:10.1186/s13062-023-00364-y)
Supplement: Supplementary file 5 — Additional file 5. Extended Protocols. [file 13062_2023_364_MOESM5_ESM.docx]

**EXTENDED MATERIALS AND METHODS**

Unless stated otherwise, all reagents were purchased from Sigma-Aldrich (St. Louis, MO) or Thermo Fisher Scientific (San Diego, CA) Life Technologies or Sigma Chemical Co.

**Tissue samples**

A retrospective study of surgical samples from patients operated at the Hospital Universitario de la Fundación Favaloro between November 2009 and December 2020, was subjected to histological diagnosis by experienced lung pathologists, informed consent was collected according to the hospital’s institutional review board (in compliance with the October 2013 Helsinki Declaration). The research protocol was approved by the Bioethics Committee of the Fundación Favaloro DDI (1473) 0719.

The histologic classification published by the World Health Organization (WHO) for tumors of the lung was applied [1].

Twenty cases of lung adenocarcinoma were analyzed and 6 cases of patients with non-small cell lung cancer not classified as adenocarcinoma were added as controls: three squamous, two neuroendocrine, and one large cell carcinoma. None of the patients had prior chemo or radiation therapy. Representative tissue blocks from each tumor-containing lesion, excluding areas with hemorrhage, necrosis, and/or fibrosis, were selected for immunohistochemical tests for AHCYL1 and Ki67 (Table 1, Figure 1B, Figure 2, and Supplemental Table 4). Also, 6 tissue samples from healthy cadaveric donors, whose organs were used for lung transplantation, died of other causes and without macroscopic or histological pulmonary pathology were added as normal controls. Sections were stained with hematoxylin and eosin to assess the histologic grade of neoplasms, which was classified according to the criteria of the College of American Pathologists (CAP). Visceral pleural invasion is categorized as absent in tumors that do not penetrate the visceral pleural elastic layer, and present when a tumor penetrates beyond the thick (external) elastic layer of the visceral pleura [2].

The AJCC and UICC TNM staging system was used for Pathologic Stage Classification for both adenocarcinoma and other non-small cell lung cancer [3].

Demographic, clinical, surgical (type of lung resection), and pathological (size, histological type, and tumor stage) patient´s data was incorporated into a database. Survival and recurrence was calculated from the time of lung resection to the date of the last consultation or the date of the patient's death.

**Immunohistochemistry staining and analyses**

Staining was performed using an automated immune stainer (BenchMark GX, Ventana Medical Systems/Roche, Tucson, AZ, USA). Briefly, formalin-fixed, paraffin embedded (FFPE) tissue sections were cut in widths of 3 µM. After deparaffinization, the slides were treated with cell conditioning reagent 1 (CC1, Roche Nr.950-124) for antigen retrieval. All primary antibodies were incubated for 32 min (5 µg/mL of anti-AHCYL1, SAB2100076, Sigma and 2 µg/mL of anti-Ki67, #790-4286, Roche). The OptiView IHC DAB Detection Kit (Roche Nr.760-700) was used for visualization in accordance with the manufacturer’s recommendations. Finally, slides were washed in distilled water, counterstained with hematoxylin (12 min) and bluing reagent (4 min), dehydrated in a descending order of alcohols, cleared in xylene, and cover-slipped Canada balsam.

The intensity of the AHCYL1 staining was scored semi-quantitatively from 0 to 3+, as follows: 0, no staining; 1, weak staining; 2, moderate staining and 3 or more, intense staining. The Ki67 antigen was used as an indicator of entry into the cell cycle of the neoplastic cells, quantifying according to the proportion of neoplastic cells with nuclei with positive staining at high magnification (400x). In all cases, the evaluations of the samples were performed independently by two pathologists (KD and JPS) blinded to the clinical characteristics of the patients. Disagreements regarding histological diagnoses and immunohistochemistry evaluation were discussed and resolved by consensus with a third pathologist (CV).

**Cell Culture**

HEK 293T, A549 and H1299 cell lines were acquired from the American Type Culture Collection (ATCC), either directly or from colleagues, kept frozen at liquid Nitrogen after received and used in culture for a maximum of 4 months. Cell culture was performed as described previously [4]. Briefly, cells lines were cultured in complete Dulbecco's Modified Eagle Medium(DMEM) supplemented with 10% fetal bovine serum (FBS), penicillin 100 U ml-1 /streptomycin 100 µg ml-1 and L-glutamine 2mM in 5% CO2 humidified atmosphere at 37 °C.

For 3D-culture assays, cells were trypsin-treated and plated in ultra-low adhesion multi-well plates (Corning, Glendale, Arizona, USA). DMEM F12 medium was supplemented with Epidermal Growth Factor (EGF) 20 ng ml-1, Fibroblast Growth Factor (FGF) 20 ng ml-1, B-27 20 µl ml-1, penicillin 100 U ml-1 / streptomycin 100 µg ml-1 and L-glutamine 2mM and changed every 2 days for 7-14 days. After 3-7 days, the number of tumorspheres was quantified using 10× magnifications under a phase contrast microscope (Carl-Zeiss, AxioVert A1 FL, Oberkochen, Germany), a Canon EOS camera and Zen pro2011; and later collected for RNA and/or protein analysis. ATCC cell lines were characterized by Short Tandem Repeat (STR) profiling. Mycoplasm contamination was evaluated monthly by PCR., and cell lines were cultured less than three months.

**Quantitative Real-time PCR**

Total RNA was extracted from cells using TRIzol Reagent and following the manufacturer’s instructions. cDNA was synthesized by using MMLV reverse transcriptase in presence of RNasin RNase inhibitor (Promega, Madison, WI, USA). Real-time PCR was performed with a Bio-Rad CFX96 Touch Real-Time PCR Detection System (Bio-Rad, CA, USA), using SYBR Green I (Roche, Tucson, AZ, USA) as a fluorescent dye. RPL19 was used as a normalization control and relative expression was calculated with the 2-ΔΔCT method [5].

Primers are listed in Supplementary Table 2.

**Cell cytometry.**

5×105 cells were fixed in ethanol 70% and stained with 50 µg/ml Propidium iodide (P1304MP, Thermo Fisher Scientific). Data were acquired on a FACS Canto II instrument (BD Biosciences) and analyzed using FlowJo software version10 (RRID:SCR_008520).

.

**Proliferation Assay**

For Resazurin staining: 2500 cells were seeded onto 96-well plates 24-hour prior to incubation. 10%FBS DMEM was supplemented with 30 µM Resazurin (#199303, Sigma) and incubated. After 2 hs, fluorescence was measured with a Multimode Plate Reader EnSpire® (Perkin-Elmer, MA, USA). For Crystal Violet Staining: 5000 cells were plated in 96-well plates in triplicate. After 24, 48, 72 and 96 h, the medium was removed, and the cells were fixed with 150 µL of methanol for 15 min. After the 96-hour fixation was complete, each well was rinsed with immersion water and cells were stained with 150 µL of a 0.06% crystal violet solution for 15 min. After incubation, excess dye was removed by immersion washing with water and the crystals were resuspended by adding 150 µL of methanol. The absorbance at 595 nm was measured and the appearance curves plotted. From these curves, the doubling time was calculated using an online tool (https://www.doubling-time.com/compute_more.php).

**Western blotting**

Cells were lysed in radioimmunoprecipitation assay buffer (RIPA) buffer with 0.1% Triton X-100 and protease inhibitor cocktail (Roche, Tucson , AZ, USA). Protein concentration was determined with Pierce BCA Protein assay kit (ThermoFisher) and 30 µg samples were prepared using Laemmli buffer. Samples were separated by SDS-PAGE and blotted onto PVDF membrane (Immobilon-P, Millipore, MA. USA). Membranes were blocked in TBS-Tween 20 (0.05%) containing 5% milk during 1 h at room temperature and incubated with specific primary antibodies overnight. Primary antibodies specific to AHCYL1 (SAB2100076, Sigma-Aldrich), POU5F1 (ab19857, Abcam, MA, USA), H3K4me3 (ab8580, Abcam), H3 (#4499, Cell Signaling, CA, USA), VEGF (Proteintech, #19003-1-AP, Planegg-Martinsried, Germany), GAPDH (ab8245, Abcam), were used. After washing with TBS-Tween 20 (0.05%), blots were incubated with HRP-conjugated secondary antibodies (Bio-Rad, Life Science) and visualized using SuperSignal West Dura kit (#34075, Thermo Scientific) following manufacturer’s instructions in GBOX-CHEMI-XT4 (Synoptics Ltd., Cambridge, UK).

**shRNA knockdown**

Knockdown cell lines were generated using Sigma Mission short hairpin RNA (shRNA) lentiviral plasmids. HEK293T cells were cotransfected with shRNA vector and lentiviral helper plasmids to obtain lentiviral particles. Particles were filtrated with a 0.45 µm pore size filter (Sartorius, Goettingen, Germany). A549 and H1299 monolayers were incubated 48 h with filtrated lentiviral particles of specific shRNAs for knockdown and a control (Mission pLKO.5-puro non-target shRNA plasmid, #SHC202). Stable cell lines were generated by Puromycin selection (3 µg.ml-1). After negative control (non-transfected A549 or H1299) died, cells were maintained in culture with 1.5 µg.ml-1 puromycin. Knockdown efficiency was confirmed by qRT-PCR and Western Blotting and periodically checked. Target sequences are listed in Supplementary Table 3.

**Limiting dilution assay**

Cells were dissociated and plated at 10, 25, 50, 100 and 200 cells/well in stem cell medium into a 96-well plate. Between 3 to 7 days after plating, the number of spheres found in each well was quantified using 10× magnifications under a phase contrast microscope (Carl-Zeiss, AxioVert A1 FL, Oberkochen, Germany) and a Canon EOS camera Tumor-initiating cell(TIC) frequency and p-values were calculated using Extreme Limiting Dilution Analysis (ELDA) software [6].

**In vivo assay**

Animals were housed with access to food and water ad libitum in ventilated mouse cages (1–5 mice per cage) at the IBioBA Animal Services Facility. Experiments were performed according to ARRIVE guidelines [7] and approved by the Ethical Committee on Animal Care and Use (CICUAL), University of Buenos Aires, Argentina (No. 110-2019) and IBioBA-CONICET (2021-03-PC). **For the mouse xenograft model** 2x10^6^ A549 or H1299 cells of each A549-NT (non-targeted) or shKD-AL1-4-cells (stably knock down of AHCYL1 clone 4) were subcutaneously injected into the right flank of NODscid mice (Jackson Laboratory, ME, USA) of 6–8 weeks of age (7-11 mice/group). Tumor growth and total animal weight were measured weekly. After 7-8 weeks, tumors were resected for analysis. Tumor volume was calculated using the following formula: 0.5 x length x width^2^ (mm^3^) [8]. **For the in vivo angiogenesis assay**, 10^6^ cells of each A549-NT or shKD-AL1-4-cells were harvested in DMEM-Trypan Blue (9:1) and were intradermally injected (27 G needle) in the right flank of male NODscid mice and the vehicle was injected in the left flank. Mice were randomly selected for each group (treatment). After 7 days, animals were sacrificed, and the skin was removed. Photographs were taken under stereo microscope (Stemi 305, Carl Zeiss, Oberkochen, Germany) using ZEN software (Carl ZEISS, Oberkochen, Germany). Small and medium vessels were measured using ImageJ software and vessel density was calculated as (number of vessels cells side − number of vessels vehicle side)/Area. Pilot experiments were used to determine sample size with adequate statistical power. Data analysis was performed by two independent blinded observers.

**Evaluation of SAH and SAM intracellular levels by UPLC-QTOF-MS**

**Chemicals**

For Liquid Chromatography−Mass Spectrometry (LC-MS) analyses: ultrapure water with 18.2 MΩ∙cm resistivity (Thermo Scientific Barnstead Micropure UV ultrapure water system, USA), Optima LC-MS-grade methanol, and ammonium acetate (Fisher Chemical, NC, USA); and analytical grade acetic acid (Fisher Chemical, NC, USA) were utilized to prepare chromatographic mobile phases, samples and chemical standard solutions. Leucine enkephalin was purchased from Waters Corp. (Milford, MA, USA). Sodium hydroxide was purchased from EMSURE®ISO (Merck Millipore, Burlington, MA, USA). S-(5'-Adenosyl)-L-methionine chloride (hydrochloride; purity ≥95%, code 13956) and S-Adenosylhomocysteine (purity ≥98%, code13603) chemical standards were purchased from Cayman Chemical (MI, USA). Sodium chloride was purchased from Anedra (Buenos Aires, Argentina).

**Cell culture for SAM and SAH extraction**

A protocol for cell culture and metabolite extraction was designed according to Lu et al. [9]. Sample generation was repeated 9 times; 4 samples for each cell line were generated with one blank in each experiment. All experiments were performed within 3 weeks of sample generation. NT and KD-AHCYL 1-4 cells with similar cell passage number (from 8 to 17) were used.

The status and monolayer confluence before seeding cells were similar for each experiment. Each cellular suspension was counted three times to minimize bias; and 1x10^6^ cells were plated for each cell line in a p100 dish. The sample process blank was generated by incubation with culture media (without cells) following the same protocol used for cells. For each experiment, one out of 4 samples was used for cell counting for data normalization [10[ as well as for protein and RNA extraction for AHCYL1 expression and AHCYL1 gen silencing monitoring; respectively. The other three samples were independently processed for SAM and SAH extraction. One of these samples was used for analytical method development and generation of quality control (QC) samples. A total of 16 samples (batch number 4 was discarded) per cell line together with blanks, a system suitability blank, and QCs were analyzed by LC-MS along two consecutive days.

After 24 h of seeding cells, the culture media was removed; cells were gently washed with fresh media, followed by 18 h incubation with the corresponding culture media. The cell seeding number was optimized to reach 80% confluence at harvest and extraction steps. Before quenching the metabolism, the media was discarded, and cell monolayers were gently and quickly washed once with 8 mL of physiological solution (NaCl 0.9% w/v) [11, 12]. Subsequently, the dish was cool-down in a water-ice bath; and immediately after, the cell metabolism was quenched using liquid nitrogen to minimize alterations [13, 14]. After nitrogen evaporation, 1 mL of cold methanol (water-ice bath) was added for metabolite extraction; and cells were harvested using a scrapper. Additional 400 µL of cold methanol was used to collect all cells from the dish. Each sample (including blanks) was frozen in liquid nitrogen; thawed, and vortex-mixed; and this cycle was repeated followed by a centrifugation step at 2000 ×g during4 min at 4 °C for cell debris removal. Supernatants were collected and stored at -20 ºC for 2 h to favor protein precipitation.[15]. After centrifugation at 15000 ×g for 15 min at 4 °C, the supernatants were collected and immediately frozen at -80 ºC after addition of ultrapure water for freezing. All samples from the same experiment were lyophilized together with process blanks to evaluate possible cross-contamination. Samples and process blanks were lyophilized in 6 batches at -80 ºC and 50 mTorr for 72 h using a Telstar LYOQuest -85 freeze-dryer (Telstar, Madrid, Spain). Frozen residues were thawed at 0 °C on a water-ice bath and reconstituted in 80 µL of 0.1% acetic acid water/methanol (90:10 v/v) solution; centrifuged at 13000 ×g for 10 min at 4°C and analyzed by ultra performance liquid chromatography quadrupole time of flight mass spectrometry (UPLC-QTOF-MS) within 6 h after sample reconstitution.

**UPLC-QTOF-MS Analyses**

SAM (S-Adenosyl methionine) and SAH intracellular levels were evaluated through a semi-targeted UPLC-QTOF-MS-based strategy, using a Waters ACQUITY UPLC I Class system fitted with a HSS T3 column (2.1 × 100 mm, 1.8 μm particle size, Waters Corporation, Milford, MA, USA), coupled to a Xevo G2S QTOF mass spectrometer (Waters Corporation, Manchester, UK) with an electrospray ionization (ESI) source. The source was operated in positive mode, the resolving power and mass accuracy of the mass spectrometer were 32000 fwhm and 0.2 ppm at m/z 556.2771 respectively. The ionic species detected for SAM and SAH were [M]+ and [M+H]+, respectively, and no in-source fragmentation was observed. A reverse phase chromatographic method was optimized using an aqueous solution with 10 mM ammonium acetate and 0.1 % acetic acid (mobile phase A), and methanol (mobile phase B). Chromatographic separation was achieved with the following gradient elution program: 0-2 min 10% B; 2-6 min 10-25% B; 6-8 min 25-65% B; 8-9 min 65-95% B; 9-13 min 95% B. The flow rate was constant at 0.20 mL. min^-1^. After each sample injection, the gradient was returned to its initial conditions in 13 min.

The injection volume was optimized for chemical standard solutions and samples (Figure 1 EXT A and B). Intra- and inter-day reproducibility was evaluated in pooled QC and study samples (Figure 1 EXT C and D). The column and autosampler tray temperatures were set at 35 and 5 °C, respectively. The mass spectrometer was operated in positive ion mode with a probe capillary voltage of 2.5 kV and a sampling cone voltage of 30 V. The source and desolvation gas temperatures were set to 120 and 300 °C, respectively. The nitrogen gas desolvation flow rate was 600 L. h^−1^, and the cone desolvation flow rate was 10 L. h^−1^. Every day, the mass spectrometer was calibrated before each batch analysis across the range of m/z 50−1200 using a 0.5 mM sodium formate solution prepared in isopropanol/water (90:10 v/v). For data drift correction during acquisition, a leucine enkephalin (m/z 556.2771) reference spray infused at 2 μL.min^−1^ every 45 s was employed.

Data acquisition was done in MS continuum mode in the range of m/z 50−1200, and the scan time was set to 1 s. Representative extracted ion chromatograms (EIC) for [SAM]+ and [SAH+H]+ at m/z 399.1451 and m/z 385.1294, respectively, were generated from samples and standard solutions (Figure 2 EXT). SAM and SAH exhibited retention times of 1.46 and 3.68 min, respectively. The comparison of experimental and simulated isotopic patterns is shown in Figure 3 EXT A and C. Additionally, UPLC-QTOF-MS/MS experiments were conducted on QC samples and standard solutions to confirm fragmentation patterns (Figure 3 EXT B and D). Product ion mass spectra were acquired with collision cell voltages between 10 and 20 V; and with two quadrupole windows, i.e., 3 Da and 1Da, to avoid ion co-selection. Ultrahigh-purity argon (≥99.999%) was used as the collision gas. Data acquisition and processing were carried out using MassLynx version 4.1 (Waters Corp., Milford, MA, USA, RRID:SCR_014271). The integration of the extracted chromatogram peak areas corresponding to SAM (m/z 399.1451) and SAH (m/z 385.1294) with a window of ±10 mDa were calculated using MassLynx version 4.1. Peak areas were normalized to the cell number and were used to perform semi quantitative analyses. Mann-Whitney U tests were used for statistical analysis, and p values < 0.05 were considered significant.

**Analytical method optimization**

Quality assurance procedures were followed before data acquisition to evaluate instrumentation and method performance. A system suitability blank (1 µM SAM and SAH standard solution, daily prepared in 0.1% acetic acid water/methanol (90:10 v/v)), a solvent blank and a process blank were analyzed at the beginning and at the end of each batch. Samples were randomly analyzed within a template of pooled QC samples that were used to verify the stability of retention times, peak shapes and areas during the analysis. Four pooled QC sample injections were performed at the beginning of each batch for conditioning the LC-MS system; the first three injections being discarded for further analysis. Two aliquots of pooled QC samples were used for analysis, one for each day of analysis, to monitor intra- and inter-day system stability and method reproducibility. Samples were randomly analyzed by UPLC-QTOF-MS with balanced classes along two consecutive days; with four samples generated in each experiment being run consecutively, and QC samples being analyzed every four study samples.

SAM and SAH were not detected either in solvent blanks or process blanks. To study metabolite stability, a 1 µM standard solution for each metabolite was monitored during 24 h at 5 ºC. No changes in peak areas were observed; neither SAH was detected in SAM standard solution, nor the opposite. Solvent volume for sample reconstitution was optimized at 80 µL to allow SAH detection, which exhibited lower intracellular levels than SAM. Sensitivity was not affected by the lyophilization process for SAM or SAH. This was evaluated by analyzing fresh and lyophilized standards at six different concentration levels and two different injection volumes (2 µL and 6 µL). All working solutions were prepared from a stock solution stored at -20 ºC for up to 2 weeks. To further explore the linear range for SAM and SAH and select the injection volume, 1 µM standard solution of each metabolite was analyzed varying injection volumes between 2 and 10 µL (Figure 1 EXT A). A similar analysis was conducted for reconstituted samples, and the injection volume was set at 6 µL for all experiments (Figure 1 EXT B). Matrix ion suppression was evaluated by comparing peak areas of two different concentration levels of standard solutions with spiked process blanks and spiked pooled QC samples. Due to the low retention time of SAM in the column, its ionization was affected by co-elution with other polar compounds and salts. Despite this limitation, it was still possible to discriminate small changes in SAM levels. No ion suppression was exhibited for SAH; and matrix composition did not affect the retention times of these compounds. To reduce organic matter deposition in the inlet of the mass spectrometer that would affect sensitivity, the eluates from the analytical column were diverted after 7 min of sample run acquisition by automatically switching the valve to waste.

The analysis of the same pooled QC sample allowed monitoring reproducibility of the method within an analytical batch and between batches (intra- and inter-day). Figure 1 EXT C shows SAM and SAH peak areas along two days of analysis for all analyzed pooled QC and study samples; and Figure 1 EXT D shows the residual standard deviation for SAM and SAH along the QC sample injections. The coefficients of variation for SAM and SAH levels detected in the consecutive injections of the pooled QC sample were below 8% intra- and inter-day, demonstrating the method reproducibility and the good quality of data. In addition, SAM and SAH peak areas in the system suitability blank were below 3% and 6%, respectively, reinforcing that the method is reproducible; and that SAM and SAH were stable during the analysis.

**Statistical analyses**

Kolmogorov-Smirnov test was performed to explore their distribution of quantitative variables. Numerical variables that presented normal distribution were displayed as mean and standard deviation. Variables with non-Gaussian distribution were presented as medians and interquartile range 25-75%. Categorical variables were reported as percentages of the total and were analyzed using Fisher's Chi-square test. For the analysis of quantitative variables with normal distribution, the t-test or ANOVA for independent samples was used. For the analysis of variables with non-parametric distribution, Mann Whitney or Kruskal-Wallis’ tests were used, as appropriate.

To assess whether there were differences in the survival rate according to demographic, clinical, surgical, and pathological variables, Ki67 staining, and intensity of immunohistochemical staining for AHCYL1, univariate Cox regression was used. Hazard ratio (HR) and confidence intervals (95% CI) were reported for each variable. All p-values reported were two-tailed and p < 0.05 was considered statistically significant. Statistical analysis was performed with SPSS 17.0 software (SPSS Inc, Chicago, Illinois).

Data were analyzed using Prism GraphPad v8.0,0 and presented as the mean and standard deviation from three independent experiments. Two-tailed Student’s t-tests and one-way ANOVA were used to define statistical significance. Progenitor frequencies from limiting dilution assays were determined using the software tool (ELDA) [6].

Statistical details of experiments can be found in the figure legends and results section.

**References**

1. WHO Classification of Tumours Editorial Board. Thoracic Tumours. 5th ed. Lyon, France: International Agency for Research on Cancer; 2021. https://publications.iarc.fr/595.

2. Schneider F, Beasley MB, Dacic S, Butnor KJ. Protocol for the Examination of Specimens From Patients With Primary Non–Small Cell Carcinoma, Small Cell Carcinoma, or Carcinoid Tumor of the Lung. Arch Pathol Lab Med. 2022;133(10):1552-1559. doi:10.5858/133.10.1552

3. Amin MB, Greene FL, Edge SB, et al. The Eighth Edition AJCC Cancer Staging Manual: Continuing to build a bridge from a population-based to a more “personalized” approach to cancer staging. CA Cancer J Clin. 2017;67(2):93-99. doi:10.3322/caac.21388

4. Ferreyra Solari NE, Belforte FS, Canedo L, et al. The NSL Chromatin-Modifying Complex Subunit KANSL2 Regulates Cancer Stem–like Properties in Glioblastoma That Contribute to Tumorigenesis. Cancer Res. 2016;76(18):5383-5394. doi:10.1158/0008-5472.CAN-15-3159

5. Livak KJ, Schmittgen TD. Analysis of Relative Gene Expression Data Using Real- Time Quantitative PCR and the 2 Ϫ ⌬⌬ C T Method. Methods. 2001;408:402-408. doi:10.1006/meth.2001.1262

6. Hu Y, Smyth GK. ELDA: Extreme limiting dilution analysis for comparing depleted and enriched populations in stem cell and other assays. J Immunol Methods. 2009;347(1-2):70-78. doi:10.1016/j.jim.2009.06.008

7. Percie du Sert N, Hurst V, Ahluwalia A, et al. The ARRIVE guidelines 2.0: Updated guidelines for reporting animal research. Boutron I, ed. PLOS Biol. 2020;18(7):e3000410. doi:10.1371/journal.pbio.3000410

8. Euhus DM, Hudd C, Laregina MC, Johnson FE. Tumor measurement in the nude mouse. J Surg Oncol. 1986;31(4):229-234. doi:10.1002/jso.2930310402

9. Lu W, Su X, Klein M, Lewis I, Fiehn O, Rabinowitz J. Metabolite Measurement: Pitfalls to avoid and practices to follow. Annu Rev Biochem. 2017;176(12):139-148. doi:10.1146/annurev-biochem-061516-044952.Metabolite

10. Muschet C, Möller G, Prehn C, de Angelis MH, Adamski J, Tokarz J. Removing the bottlenecks of cell culture metabolomics: fast normalization procedure, correlation of metabolites to cell number, and impact of the cell harvesting method. Metabolomics. 2016;12(10). doi:10.1007/s11306-016-1104-8

11. Dietmair S, Timmins NE, Gray PP, Nielsen LK, Krömer JO. Towards quantitative metabolomics of mammalian cells: Development of a metabolite extraction protocol. Anal Biochem. 2010;404(2):155-164. doi:10.1016/j.ab.2010.04.031

12. Kapoore RV, Coyle R, Staton CA, Brown NJ, Vaidyanathan S. Influence of washing and quenching in profiling the metabolome of adherent mammalian cells: A case study with the metastatic breast cancer cell line MDA-MB-231. Analyst. 2017;142(11):2038-2049. doi:10.1039/c7an00207f

13. Lorenz MA, Burant CF, Kennedy RT. Reducing time and increasing sensitivity in sample preparation for adherent mammalian cell metabolomics. Anal Chem. 2011;83(9):3406-3414. doi:10.1021/ac103313x

14. Bi H, Krausz KW, Manna SK, Li F, Johnson CH, Gonzalez FJ. Optimization of harvesting, extraction, and analytical protocols for UPLC-ESI-MS-based metabolomic analysis of adherent mammalian cancer cells. Anal Bioanal Chem. 2013;405(15):5279-5289. doi:10.1007/s00216-013-6927-9

15. Sarafian MH, Gaudin M, Lewis MR, et al. Objective set of criteria for optimization of sample preparation procedures for ultra-high throughput untargeted blood plasma lipid profiling by ultra performance liquid chromatography-mass spectrometry. Anal Chem. 2014;86(12):5766-5774. doi:10.1021/ac500317c


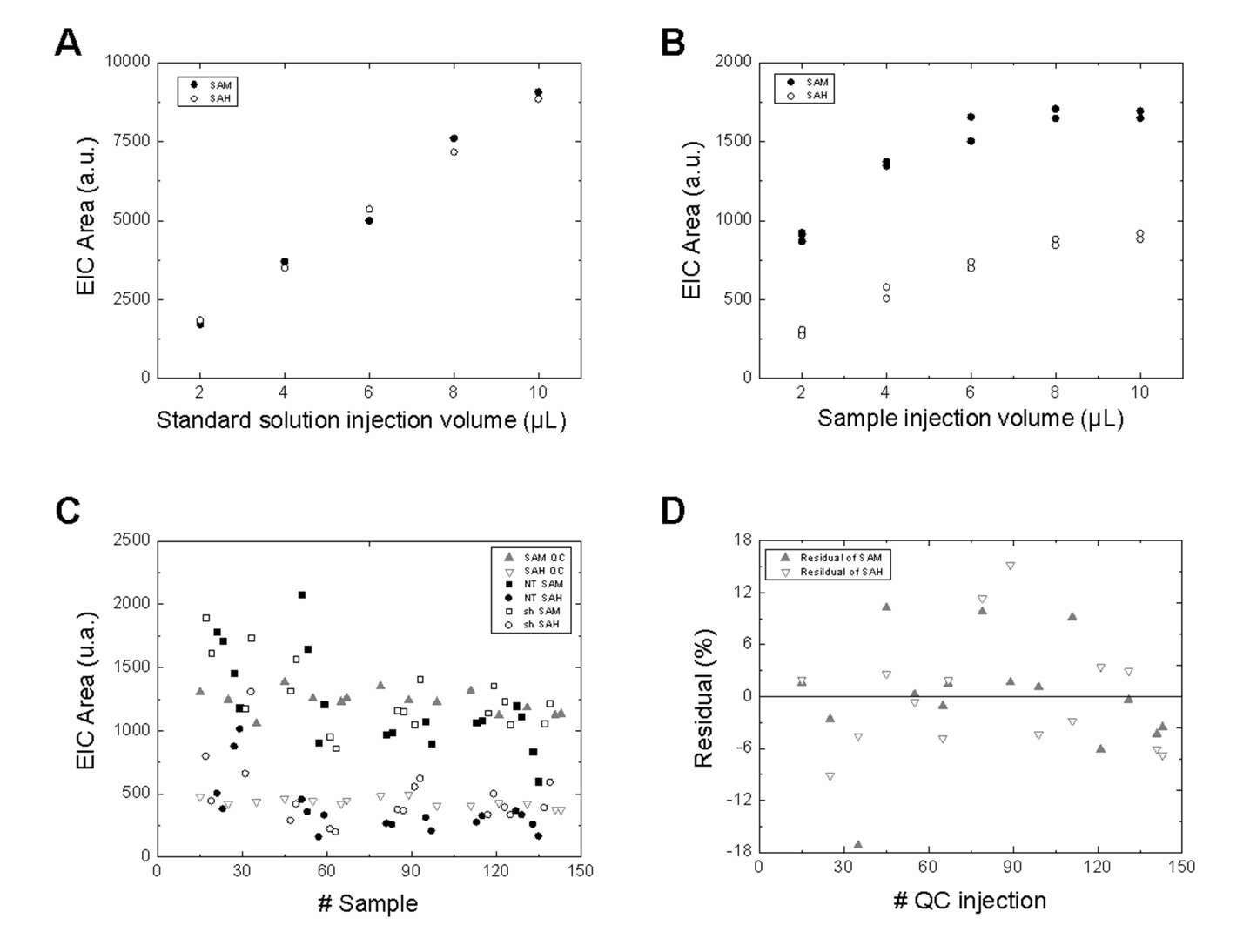


**Figure 1 EXT**. Peak areas for SAM and SAH for a 1 µM standard solution (A) and for a sample (B) for different injection volumes from to 2 to 10 µL. Intra- and inter-day evaluation of the method reproducibility through the analysis of SAM and SAH peak areas in pooled QC and study samples (C). Residual standard deviation of SAM and SAH peak areas as a function of the pooled QC injection order (D).


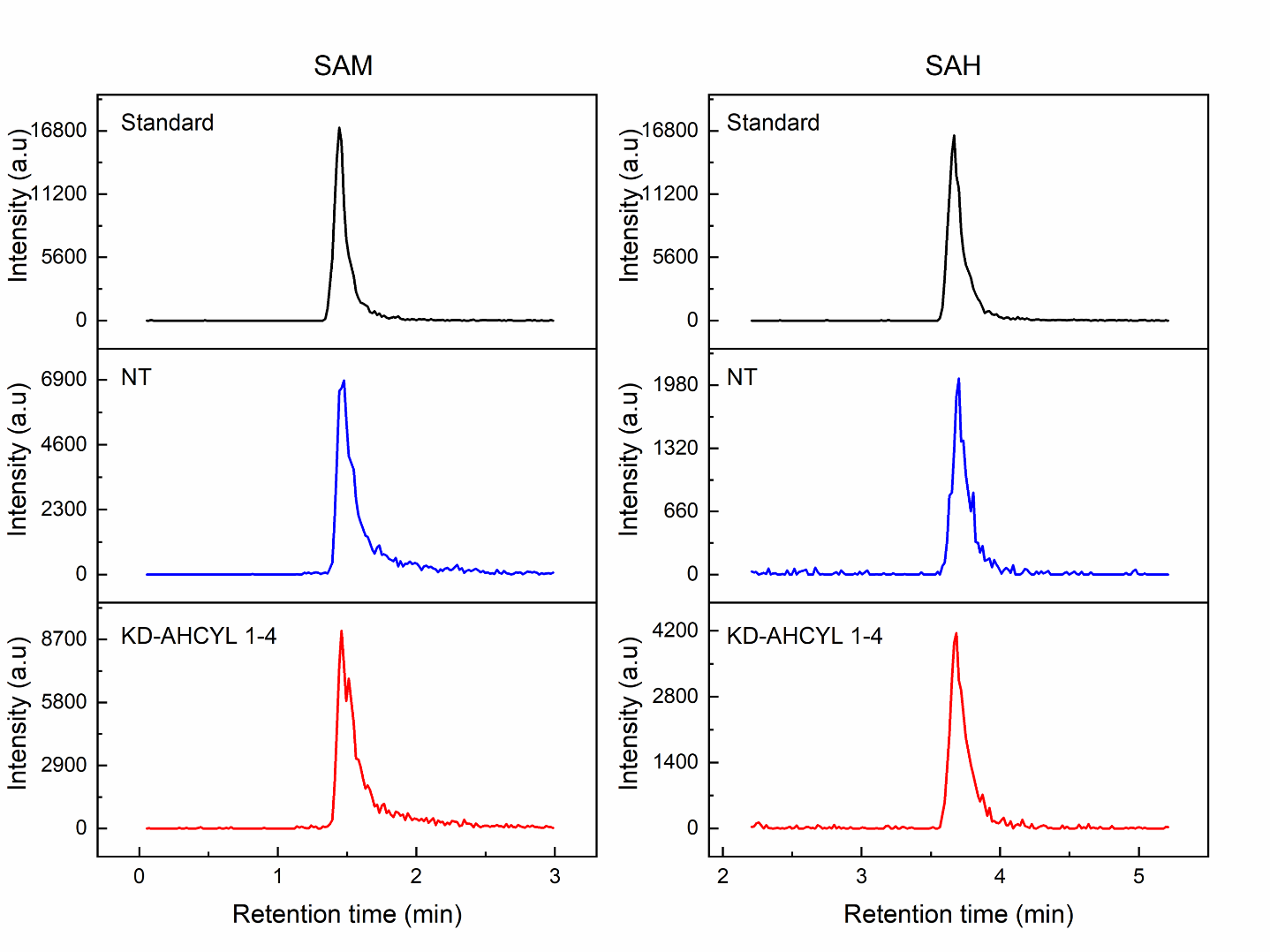


**Figure 2 EXT**. Representative extracted ion chromatograms (EIC) for [SAM]+ and [SAH+H]+ at m/z 399.1451 and m/z 385.1294, respectively, were generated from standard solutions, NT, and KD-AHCYL 1-4 samples.





**Figure 3 EXT**. A) Mass spectrum for [SAM]+ ion with m/z 399.1451 detected in a pooled QC sample (green), and its simulated isotopic pattern for [C15H23N6O5S]+ (blue); B) Product ion mass spectra of [C15H23N6O5S]+ precursor ion for a pooled sample and for a 1 µM SAM standard solution (blue) using a collision cell voltage of 10 V and a quadrupole window of 1 Da (green) to avoid co-selection; C) Mass spectrum for [SAH+H]+ ion with m/z 385.1294 in a pooled QC sample (green) and its simulated isotopic pattern for [C14H20N6O5S+H]+; D) Product ion mass spectra of [C14H20N6O5S+H]+ precursor ion for a pooled QC sample and for a 1 µM SAH standard solution (blue) using a collision cell voltage of 10 V and a quadrupole window of 1 Da (green) to avoid co-selection.
