# Supplementary material for: S-adenosylhomocysteine hydrolase-like protein 1 (AHCYL1) inhibits lung cancer tumorigenesis by regulating cell plasticity
Source: Biol Direct. 2023 Mar 5;18:8. doi: 10.1186/s13062-023-00364-y (PMC9985837; doi:10.1186/s13062-023-00364-y)
Supplement: Supplementary file 6 — Additional file 6. Table S4. Clinical and histopathological data from patients with lung cancer. [file 13062_2023_364_MOESM6_ESM.doc]

**Supplementary Table 4**. Clinical and histopathological data from patients with lung cancer.

|  |  |  |  |  |  |  |  |  |  |  |  |  |  |
| --- | --- | --- | --- | --- | --- | --- | --- | --- | --- | --- | --- | --- | --- |
| **Type of**  **tumor** | **Case n°** | **AHCYL1** | **Ki 67** | **Gender** | **Age**  **(years)** | **Follow-up time**  **(days)** | **Alive** | **Recurrence** | **pTNM** | **UICC**  **stage** | **Size**  **(mm)** | **Pleural**  **infiltration** | **Histologic**  **grade** |
| LUAD | 1 | 2 | 30 | M | 53 | 3294 | Y | N | pT2a pN0 | IB | 35 | Y | 2 |
| LUAD | 2 | 4 | 5 | F | 57 | 1694 | Y | N | pT1B pN0 | IA | 16 | N | 2 |
| LUAD | 3 | 3 | 1 | M | 75 | 3687 | Y | N | pT1B pN0 | IA | 12 | N | 2 |
| LUAD | 4 | 4 | 10 | F | 70 | 2822 | Y | N | pT1A pNx | IA | 16 | N | 1 |
| LUAD | 5 | 3 | 5 | F | 76 | 1680 | N | N | pT1B pN0 | IA | 22 | N | 3 |
| LUAD | 6 | 3 | 5 | M | 55 | 2759 | Y | N | pT2a pN0 | IB | 25 | Y | 3 |
| LUAD | 7 | 4 | 5 | M | 63 | 1274 | N | N | pT4 pN1 | IIIA | 22 | N | 3 |
| LUAD | 8 | 4 | 15 | F | 57 | 2285 | Y | N | pT2b pN0 | IB | 36 | N | 1 |
| LUAD | 9 | 4 | 5 | M | 67 | 2312 | Y | Y | pT2b pN0 | IIA | 62 | Y | 2 |
| LUAD | 10 | 4 | 5 | F | 70 | 2258 | Y | N | pT4 pN0 | IIIA | 35 | N | 2 |
| LUAD | 11 | 3 | 5 | F | 54 | 9 | Y | N | pT1A pN0 | IA | 8 | N | 3 |
| LUAD | 12 | 3 | 20 | M | 75 | 1298 | N | N | pT2a pN1 | IIA | 47 | Y | 2 |
| LUAD | 13 | 2 | 70 | M | 72 | 379 | N | Y | pT1b pNx | IA | 13 | N | 2 |
| LUAD | 14 | 3 | 45 | M | 42 | 2962 | Y | N | pT2a pN0 | IB | 34 | N | 3 |
| LUAD | 15 | 1 | 85 | M | 70 | 5 | N | N | pT3 pN1 | IIIA | 26 | Y | 3 |
| LUAD | 16 | 2 | 60 | M | 72 | 365 | Y | N | pT1b pN1 | IB | 15 | N | 3 |
| LUAD | 17 | 3 | 15 | F | 80 | 382 | N | N | pT2a pN0 | IB | 29 | Y | 2 |
| LUAD | 18 | 3 | 40 | F | 55 | 1219 | Y | Y | pT2a pN1 | IIB | 33 | Y | 3 |
| LUAD | 19 | 3 | 21 | F | 66 | 1016 | Y | N | pT3 pN0 | IIB | 73 | Y | 1 |
| LUAD | 20 | 2 | 20 | M | 70 | 833 | Y | N | pT2a pN0 | IB | 32 | N | 3 |
| LSCC | 21 | 2 | 90 | M | 66 | 554 | Y | N | pT1c pN0 | IA3 | 25 | N | 2 |
| LCNC | 22 | 3 | 95 | F | 73 | 351 | N | Y | pT2a pN1 | IIB | 23 | Y | 3 |
| LSCC | 23 | 4 | 40 | M | 58 | 30 | N | N | pT1B pN0 | IB | 36 | N | 2 |
| LCC | 24 | 2 | 68 | M | 60 | 9 | N | N | pT4 pN1 | IIIA | 73 | N | 4 |
| LSCC | 25 | 2 | 65 | F | 76 | 904 | Y | Y | pT1b pN0 | IA2 | 18 | N | 3 |
| LCNC | 26 | 1 | 77 | M | 66 | 3 | Y | N | pT4 pN0 | IIIA | 128 | Y | 3 |

LUAD,lung adenocarcinoma; LSCC, lung squamous cell carcinoma;LCNC,large cell neuroendocrine carcinoma;LCC,large cell carcinoma; M, male; F, female; Alive (Y, yes; N,no);

Recurrence (Y, yes; N, no); Pleural infiltration (Y, yes; N, no).
