# Supplementary material for: S-adenosylhomocysteine hydrolase-like protein 1 (AHCYL1) inhibits lung cancer tumorigenesis by regulating cell plasticity
Source: Biol Direct. 2023 Mar 5;18:8. doi: 10.1186/s13062-023-00364-y (PMC9985837; doi:10.1186/s13062-023-00364-y)
Supplement: Supplementary file 7 — Additional file 7. Table S5. Univariate Cox regression to compare survival in patients with lung adenocarcinoma. [file 13062_2023_364_MOESM7_ESM.docx]

**Supplementary Table 5. Univariate Cox regression to compare survival in patients with lung adenocarcinoma.**

| **Variable** | **Cox Regression** | |
| --- | --- | --- |
|  | ***Hazard Ratio* (95%IC)** | ***P value*** |
| Age (years) | 1.174 (0.995-1.323) | 0.059 |
| Gender (male) | 1.744 (0.319-9.544) | 0.522 |
| Histological grade | 2.118 (0.574-7.822) | 0.260 |
| UICC TNM stage | 1.319 (0.763-2.280) | 0.321 |
| Ki67 | 1.048 (1.007-1.090) | 0.022* |
| AHCYL1 | 0.169 (0.033-0.869) | 0.033* |
| *corresponds to statistically significant differences (*p*<0.05**)** | | |
